# Systematic review and meta-analysis of interventions to increase the uptake of vaccines recommended during pregnancy

**DOI:** 10.1038/s41541-025-01120-1

**Published:** 2025-04-19

**Authors:** Annette K. Regan, Honorine Uwimana, Stacey L. Rowe, Elizabeth Jitka Olsanska, Brianna Agnew, Eliana Castillo, Alice Fiddian-Green, Michelle L. Giles

**Affiliations:** 1 Department of Research & Evaluation, Kaiser Permanente Southern California, Pasadena, CA USA; 2 Fielding School of Public Health, University of California Los Angeles, Los Angeles, CA USA; 3 School of Nursing and Health Professions, University of San Francisco, San Francisco, CA USA; 4 College of Arts & Sciences, University of San Francisco, San Francisco, CA USA; 5 Departments of Medicine and Obstetrics & Gynaecology, University of Calgary, Calgary, AB Canada; 6 Department of Obstetrics and Gynecology, Monash University, Melbourne, VIC Australia

**Keywords:** Epidemiology, Infectious diseases

## Abstract

Although immunization during pregnancy can protect mothers and their infants from vaccine-preventable morbidity and mortality, vaccination rates are often poor. We systematically reviewed the literature from inception to July 4, 2023, for randomized and non-randomized quasi-experimental studies estimating the effects of interventions to increase vaccination during pregnancy. Of 9331 studies screened, 36 met inclusion criteria, including 18 demand-side interventions, 11 supply-side interventions, and seven multi-level (demand and supply-side) interventions. Demand-side interventions commonly addressed patient education, showing modest improvement (pooled RR 1.18; 95% CI: 1.04, 1.33; *I*^2^ = 63.1%, low certainty). Supply-side interventions commonly implemented Assessment-Feedback-Incentive-eXchange interventions with little improvement (pooled RR 1.13; 95% CI: 0.96, 1.33; *I*^2^ = 94.0%, low certainty). Multi-level interventions were modestly effective in increasing vaccination (pooled RR 1.62; 95% CI: 1.09, 2.42; *I*^2^ = 97%, very low certainty). Interventions identified in the literature mostly resulted in low to moderate increases in vaccination with likely high heterogeneity and low to very low certainty in the findings.

## Introduction

Pregnancy and early infancy present life stages susceptible to more severe infection^1^. Additionally, infants under the age of six months are either too young to receive licensed vaccines (e.g., influenza and COVID-19 vaccines) or are too young to have achieved a protective level of antibodies in response to their first few doses of routine childhood vaccines (e.g., pertussis and tetanus-containing vaccines). Maternal immunization, or vaccination during pregnancy, can prevent severe illness both in the mother and the newborn^2,3^. Maternal antibodies offer direct protection against illness to the immunized mothers, and passive protection to the newborn through transplacental antibody passage^4^. In some cases (e.g., respiratory syncytial virus vaccine), maternal immunization may offer the only vaccine strategy to protect infants from disease.

Several vaccines are routinely recommended during pregnancy, including influenza, COVID-19, pertussis and tetanus-containing vaccines, and respiratory syncytial virus (RSV) vaccine. Additional vaccines may be recommended in specific circumstances, such as pneumococcal, meningococcal, *Haemophilus influenzae* type b, hepatitis A and B, inactivated polio, typhoid, yellow fever, Japanese encephalitis, rabies, anthrax, Ebola, dengue, and smallpox vaccines^5,6^. Presently, there are additional vaccines under development for future use during pregnancy to prevent maternal-newborn infection, including group B streptococcus^5^.

Routinely recommended vaccines have been shown to be safe and effective during pregnancy. For example, pertussis vaccination during pregnancy prevents between 69-91% of pertussis infections and up to 94% of pertussis-associated hospitalizations and deaths among infants < 2-months-old^2^. Influenza vaccination during pregnancy prevents between 30% and 63% of influenza infections among infants <6 -months-old and 31–70% of influenza infections among pregnant persons^4^.

Despite the benefits of immunization, low vaccination rates during pregnancy are commonly documented in high-income and low and middle-income countries^7–10^, indicating that interventions to increase vaccine uptake during pregnancy are needed. Vaccine uptake is driven by a combination of patient and provider factors, including patient awareness and acceptance of vaccines and access to and affordability of vaccines^11^. Interventions to increase vaccine uptake can therefore address demand-side factors (i.e., patient awareness and acceptance), supply-side factors (i.e., increased access to vaccines by enhanced immunization services), or a combination of both^12^. This review aimed to systematically describe and assess the effects of interventions designed to increase uptake of recommended vaccines among pregnant persons.

## Results

**Fig. 1:**
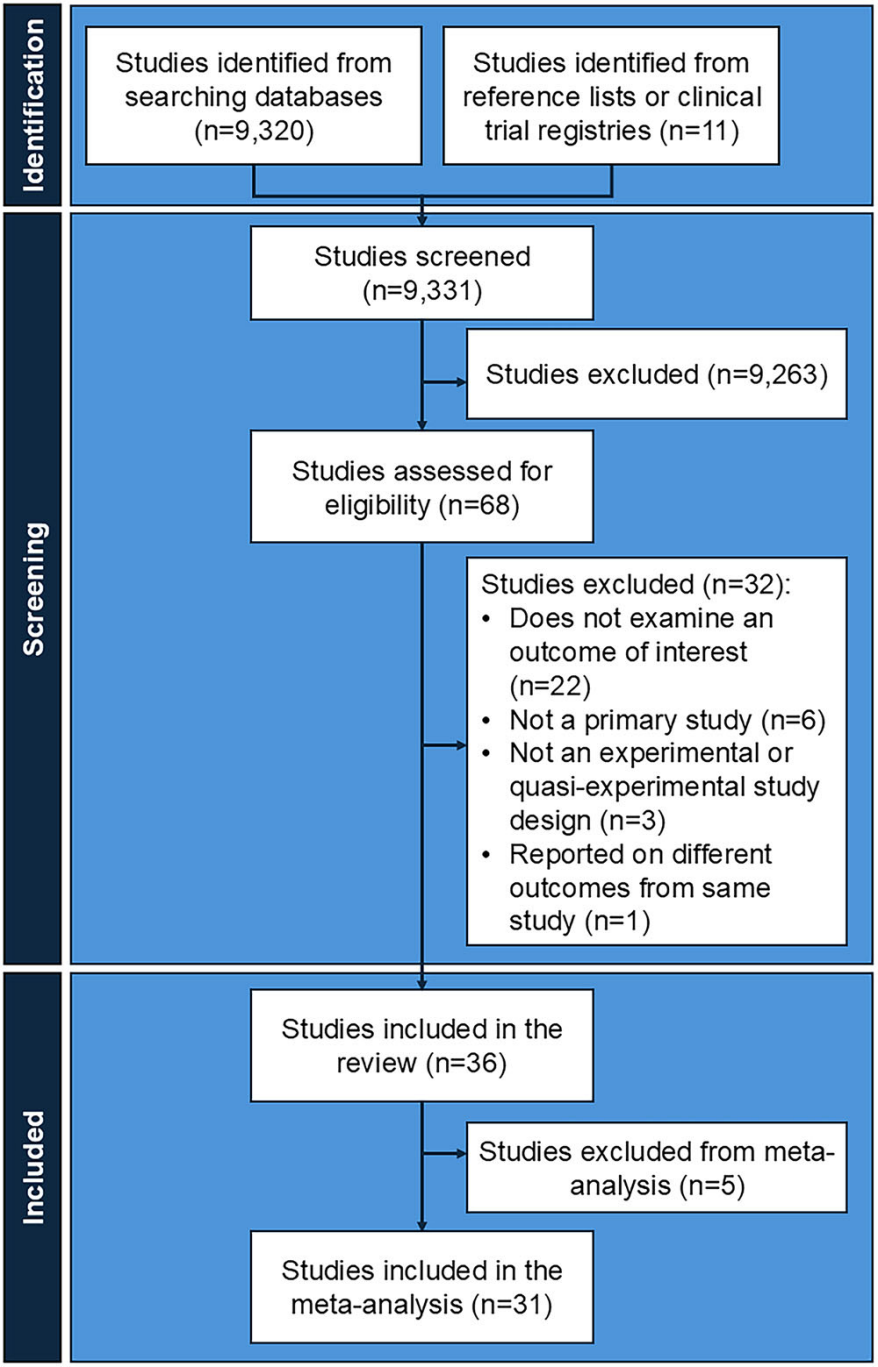
Selection of studies. PRISMA diagram for selection of studies evaluating the effect of interventions to increase the uptake of vaccines recommended during pregnancy.

A total of 14,372 studies were identified from database searches, and 11 studies were identified from review of reference lists and clinical trial registries. After removing duplicates, the abstracts and titles of 9331 studies were screened for inclusion, of which 68 studies met inclusion criteria. After reviewing 68 full-text articles, 36 unique studies met inclusion criteria and 32 were suitable for meta-analysis (Fig. 1). A summary of full-text articles excluded from further reviews is provided in Table S1. Eighteen (50%) studies were randomized trials, including 15 randomized controlled trials and three cluster randomized controlled trials. We identified one non-randomized controlled trial^13^. Seventeen (47%) studies were quasi-experimental, including ten studies using a historical control, six studies using a geographic control, and one study using both a historical and geographic control. Fourteen studies aimed to increase uptake of influenza vaccine, four to increase uptake of pertussis-containing vaccines, and six to increase uptake of influenza and pertussis-containing vaccines. Eleven studies aimed to increase uptake of the tetanus toxoid vaccine, and one study aimed to increase COVID-19 vaccine uptake.

Nineteen (53%) of the 36 included studies were conducted in the United States (Table 1). The remaining studies were conducted in one of 14 other countries; 12 (34%) in a low or middle-income country (LMIC) and 24 (67%) in a high-income country (HIC; Fig. S1). We identified 24 studies evaluating patient-level (demand-side) interventions and 17 studies that evaluated systems or provider-level (supply-side) interventions; 19 studies implemented patient-level interventions only^13–30,38^, 11 studies implemented systems or provider-level interventions exclusively^31–41^, and six were bundled with interventions that targeting patients and providers or health systems^42–47^.

**Table 1:** Characteristics of studies reporting on interventions to increase the uptake of vaccines recommended during pregnancy, by vaccine

Author, year	Study design	Study funding	Study location	Study setting	Sample	Intervention	Comparison	Outcome(s) assessed
COVID-19 vaccine (*n* = 1)								
Momani et al.^13^^*^	Non-randomized controlled trial	Applied Science Private University, Amman, Jordan	Jordan	Prenatal/maternal government, private, and educational health sector prenatal/maternal clinics within three of the 12 governorates in Jordan (Amman, Irbid, and Zarqa)	190 currently pregnant people	Patient-level: patient education	Usual care	(1) COVID-19 vaccine uptake; (2) COVID-19 vaccine hesitancy
Influenza vaccine (*n* = 14)								
Dehlinger et al.^42^	Quasi-experimental with a historical control	No information provided	Midwest, USA	Three women’s health ambulatory clinics within an urban, Midwestern health care system	2967 pregnant people	Provider-level: provider education, provider reminders Patient-level: patient education	Pre-intervention period	(1) Influenza vaccine uptake; (2) proportion of influenza vaccines recorded in EHR; (3) number of patient records without an associated immunization code
Frew et al.^14^	Randomized controlled trial	Centers for Disease Control and Prevention (grant # 5P01TP000300)	Georgia, USA	Four OB-GYN clinics	95 pregnant people	Patient-level: patient education + persuasive messaging	Usual care	(1) Influenza vaccine uptake; (2) likelihood of vaccinating in future pregnancy; (3) reasons for not getting vaccinated
Goodman et al.^15^	Randomized controlled trial	Cleveland Clinic Research Committees	Ohio, USA	Three suburban Cleveland Clinic Health System OB-GYN offices	105 pregnant people	Patient-level: patient education	Hand hygiene patient education intervention	(1) influenza vaccine uptake; (2) vaccine health beliefs
Jordan et al.^16^	Randomized controlled trial	No specific funding obtained for study (initial study sponsored by Johnson & Johnson)	National, USA	National online study	3905 pregnant people	Patient-level: patient education; patient reminders	Standard educational message	(1) influenza vaccine uptake; (2) future vaccine intentions
Klatt and Hopp et al.^31^	Quasi-experimental with historical control	No information provided	Wisconsin, USA	OB-GYN clinic	640 pregnant people	Provider-level: provider reminder	Pre-intervention period	(1) influenza vaccine uptake; (2) reasons for no vaccination
Meharry et al. 2013	Randomized controlled trial	Sigma Theta Tau	Northeastern, USA	Hospital-based prenatal care clinics in Connecticut, USA	135 pregnant people	Patient-level: patient education	Usual care	(1) Influenza vaccine uptake; (2) influenza vaccine health beliefs
Mouzoon et al.^32^	Quasi-experimental with historical control	Kelsey Research Foundation	Texas, USA	Large multispecialty medical organization covering 19 clinics in Houston Texas	11,420 electronic health records	Systems-level: immunization champion; standing orders; AFIX Provider-level: provider education	Pre-intervention period	(1) Influenza vaccine uptake; (2) uptake among those with chronic conditions; (3) uptake by gestational age
Moniz et al.^18^	Randomized controlled trial	Amy Roberts Health Promotion Foundation	Pennsylvania, USA	Outpatient OB-GYN clinic at a large academic hospital	204 pregnant people	Patient-level: patient education	General preventive health messages	(1) Influenza vaccine uptake; (2) preventive health beliefs
Parsons et al. 2022*	Quasi-experimental with historical control	Coventry University	England, UK	Online panel	411 pregnant people	Patient-level: patient education	Pre-intervention	(1) Influenza vaccine intent; (2) influenza vaccine health beliefs
Regan et al.^20^	Randomized controlled trial	Department of Health, Western Australia	Western Australia, Australia	Ten general practice clinics in Western Australia	239 pregnant people	Patient-level: patient reminder	Usual care	Influenza vaccine uptake
Stockwell et al.^21^	Randomized controlled trial	DHHS Health Resources and Services Administration Maternal and Child Health Bureau (R40MC17169)	New York City, USA	Five community-based multispecialty clinics in NYC	1187 pregnant people	Patient-level: patient reminder	Usual care	Influenza vaccine uptake
Wong et al.^22^	Randomized controlled trial	Health and Medical Research Fund Government of the Hong Kong Special Administration Region (Hong Kong SAR, Grant # 12111272)	Hong Kong	Four public hospital-based antenatal care clinics	321 pregnant people	Patient-level: patient education	Usual care	(1) Influenza vaccine uptake; (2) initiation of influenza vaccine conversation with healthcare professional
Wootton et al.^48^	Randomized controlled trial	KL2 CCTS Supplement Award (grant # 3KL2RR024149-0551), a CTSA Award (grant # UL1RR024148), and Larry C. Gilstrap Center for Perinatal and Women’s Health Research (University of Texas Health Science Center)	Texas, USA	2 hospital-based OB-GYN clinics	280 pregnant people	Systems-level: opt-out delivery	Usual care	Influenza vaccine uptake
Yudin et al.^23^	Randomized controlled trial	St. Michael’s Hospital Innovation Fund Grant (grant # SMHAIF-078)	Toronto, Canada	Hospital-based antenatal clinic	317 pregnant people	Patient-level: patient education	Usual care	Influenza vaccine uptake
Pertussis-containing vaccines (*n* = 4)								
Kriss et al.^24^	Randomized controlled trial	No information provided	Georgia, USA	Four OB-GYN clinics	106 Black pregnant people	Patient-level: patient education + persuasive messaging	Usual care	(1) Tdap vaccine uptake during pregnancy or immediately postpartum; (2) intention to receive Tdap in future pregnancy; (3) reasons for no Tdap vaccination
Li et al.^33^	Quasi-experimental with geographic control	Quebec Ministry of Health and Social Services	Quebec, Canada	Four Quebec health regions	946 pregnant people	Systems-level: models of vaccine delivery	Usual care (community health clinics)	Tdap vaccine uptake
Mazzoni et al.^43^	Quasi-experimental with historical control	Centers for Disease Control and Prevention (grant # IP000501-03)	Colorado, USA	Two community-based OB-GYN clinics	2,650 pregnant people	Systems-level: standing orders; vaccines on-site; immunization champion; non-provider staff education; AFIX Provider-level: provider education; provider feedback Patient-level: patient education	Pre-intervention period	Tdap vaccine uptake
Payakachat et al.^25^	Randomized controlled trial	University of Arkansas Medical Sciences College of Pharmacy Summer Research Fellowship Program, National Institute of Mental Health (grant # R03MH102495)	Arkansas, USA	Women’s clinics at an academic medical center	279 pregnant people	Patient-level: patient education	Usual care	(1) Tdap vaccine uptake; (2) influenza vaccine health beliefs
Influenza & pertussis-containing vaccines (*n* = 6)								
Brewer et al.^44^	Quasi-experimental with historical control	Centers for Disease Control and Prevention (grant # IP000501-03)	Colorado, USA	Five private OB-GYN clinics	275 OB-GYN medical charts	Systems-level: standing orders; provision of vaccines on-site; enhanced vaccine documentation; immunization champion; non-provider staff education; AFIX Provider-level: provider education; provider feedback Patient-level: patient education	Pre-intervention period	Documentation of (1) historical vaccination; (2) vaccine eligibility; (3) vaccine administration in office; (4) vaccine refusal; (5) and placement of vaccine information in EHR
Chamberlain et al.^45^; Chamberlain et al. 2016	Cluster randomized controlled trial	Centers for Disease Control and Prevention (grant no. 5P01TP0003000)	Georgia, USA	Eleven OB-GYN practices	325 pregnant people	Systems-level: Immunization champion Provider-level: provider education Patient-level: patient education; map to local vaccine sites	Usual care	(1) Influenza vaccine uptake; (2) Tdap vaccine uptake; (3) vaccine knowledge, attitudes, and beliefs; (4) maternal and infant vaccine hesitancy; (5) intent to receive influenza or pertussis vaccine; (6) intent to vaccinate infant
Dudley et al.^29^^*^	Randomized controlled trial	National Institute for Allergy and Infectious Diseases at the National Institutes of Health (grant # R01AI110482)	Colorado and Georgia, USA	Eleven OB-GYN practices	2,087 pregnant people	Patient-level: patient education	Usual care	(1) Vaccine knowledge, attitudes, and behaviors; (2) pro-vaccine social norms; (4) trust in vaccine information
O’Leary et al.^26^	Randomized controlled trial	Agency for Healthcare Research and Quality (R01HS021492)	Colorado, USA	Integrated Health System	1093 pregnant people	Patient-level: patient education	Usual care	(1) Influenza vaccine uptake; (2) Tdap vaccine uptake
Omer et al.^46^	Cluster and individually- randomized controlled trial	National Institute for Allergy and Infectious Diseases at the National Institutes of Health (grant # R01AI110482)	Georgia and Colorado, USA	24 OB-GYN practices	2200 pregnant people	Systems-level: standing orders; immunization champion; enhanced vaccine documentation; AFIX Provider-level: provider education Patient-level: patient education; map to local vaccine sites	Usual care	(1) Influenza vaccine uptake; (2) Tdap vaccine uptake
Spina et al.^34^	Quasi-experimental with historical control	National Institute for Allergy and Infectious Diseases at the National Institutes of Health (grant # R01AI110482)	Colorado and Georgia, USA	11 OB-GYN practices in two US states	889 OB-GYN medical charts	Systems-level: immunization champion; standing orders; enhanced vaccine documentation; AFIX Provider-level: provider education	Pre-intervention period	(1) Influenza vaccine uptake; (2) Tdap vaccine uptake
Tetanus toxoid vaccine (*n* = 11)								
Balakrishnan et al.^35^	Quasi-experimental with a historical and geographic control	Bill and Melinda Gates Foundation (via Care-India)	Bihar, India	District-wide implementation of intervention with frontline community health workers	512 frontline community health workers	Systems-level: mHealth service (continuum of care; enhanced vaccine documentation) Provider-level: provider reminder	(1) Neighboring region; (2) Pre-intervention period	(1) Early registration of pregnancy; (2) complete 3+ antenatal care visits; (3) received 1+ tetanus toxoid vaccines; (4) received 90+ iron and folic acid tablets; (5) institutional delivery; (6) initiation of breastfeeding within 1 h of birth; (7) receipt of 1+ postnatal home visit
Bonfrer et al.^36^	Quasi-experimental with a historical control	No information provided	11 provinces, Burundi	Country-wide implementation of the intervention by the Ministry of Health	700 healthcare facilities;	Systems-level: performance-based financing	Pre-intervention period	(1) Institutional delivery; (2) 1+ antenatal care visit; (3) 1+ tetanus toxoid vaccination; (4) 1+ infant vaccination; (5) BCG vaccination; (6) modern family planning among women 15-49 years; (7) household use of 1+ bed net; (8) quality of healthcare
Chakrabarti et al.^27^	Quasi-experimental with a geographic control	No relevant funding to disclose	Odisha, India	All regions in Odisha, India	208,895 mother-child dyads	Patient-level: conditional cash transfer	Neighboring region	(1) 4+ antenatal care visits; (2) 100+ iron-folic acid tablets during pregnancy; (3) 2+ tetanus toxoid vaccinations during pregnancy; (4) receipt of breastfeeding counseling; (5) child fully immunized; (6) vitamin A supplementation in child; (7) stunting prevalence; (8) pediatric anemia prevalence; (9) maternal anemia prevalence
Choudhury et al.^30^^*^	Quasi-experimental with a geographic control	Simavi, the Netherlands (grant # 3312005) and Deutsche Welthungerhilfe (grant # WHHInd/1287)	India	2 rural villages	1480 pregnant people	Patient-level: patient education	Neighboring region	Awareness of the need for antenatal care, tetanus toxoid vaccination, iron supplementation, danger signs in pregnancy, danger signs in labor, and hygiene (“5 cleans”)
de Walque et al.^40^	Quasi-experimental with a geographic control	World Bank Health Results Innovation Trust Funds (HRITF)	Cameroon	206 healthcare facilities in participating regions	2971 household members	Systems-level: performance-based financing	Neighboring region	(1) Skilled delivery; (2) 2+ ANC visits; (3) 1+ tetanus toxoid vaccination during pregnancy; (4) 1+ postnatal care visit; (5) modern contraceptive use (6) receipt of childhood vaccines; (8) healthcare spending; (9) patient satisfaction with antenatal care; (10) availability of facility staff, equipment, and supplies
Mwase et al.^37^	Cluster randomized controlled trial	World Bank Health Results Innovation Trust Funds (HRITF)	Burkina Faso	Rural health districts	11,010 women of reproductive age (15-49 years)	Systems level: Performance-based financing	Usual care	(1) Antenatal care in the first trimester; (2) 4+ antenatal care visits; (3) 2+ doses of tetanus toxoid vaccine in pregnancy; (4) HIV testing in pregnancy; (5) iron supplementation; (6) facility-based delivery; (7) 1+ postnatal care visit; (8) 3+ postnatal care visits; (9) modern family planning
Ohly et al. 2018	Quasi-experimental with historical control	UK Department for International Development (grant # GPAF-INN-04)	Pakistan	Rural brick kiln community in the Peshawar District, Khyber Pakhtunkhwa Province	1238 pregnant people	Systems-level: service delivery; staff capacity development; community engagement Patient-level: conditional cash transfer	Pre-intervention	(1) Satisfaction with healthcare; (2) use of family planning; (3) women following a birth preparedness plan; (4) receipt of 1+ tetanus toxoid vaccine during pregnancy; (5) unskilled delivery; (6) problems during delivery; (7) children <5 years vaccinated with BCG; (8) households with animal in home
Sato and Fintan^28^	Randomized controlled trial	Institute for Research on Women & Gender, the Rackham Graduate School, the Department of Afro-American and African Studies, the Department of Economics, and the Center for the Education of Women at the University of Michigan; the Japan Society for the Promotion of Science (grant # 22223003), and the Yamada Scholarship Foundation	Nigeria	Catchment areas for 10 health clinics within Adamawa State/Rural northern Nigeria	2482 pregnant and non-pregnant women	Patient-level: conditional cash transfer	Small conditional cash transfer	Receipt of 1+ tetanus toxoid vaccine
Shah et al.^38^	Quasi-experimental with historical control	United Nations Population Fund, United States Agency for International Development, and the Indian Council of Medical Research	Egypt, Philippines	Thirteen health centers across the eight countries	1783 pregnant people	Patient-level: home-based health record	Usual care	(1) Use of home-based maternal health record; (2) 1+ antenatal care visit; (3) 1+ postnatal care visit; (4) women receiving family planning education; (5) receipt of 1+ tetanus toxoid vaccine; (6) number of infant growth charts made; (7) number of women identified as at-risk; (8) number of at-risk women referred; (9) number of women cared for at referral centers; (10) number of records giving feedback from referral centers
Woolley et al.^39^	Quasi-experimental with geographic control	Atlantic Philanthropies	Philippines	Five communities in the Zamboanga	827 recent mothers of a child 0–5 years	Provider-level: provider education	Usual care	(1) receipt of 4+ antenatal care visits; (2) first antenatal care visit in 1st trimester; (3) mother received information about complications; (4) receipt of 1+ tetanus toxoid vaccine during pregnancy; (5) weight measured during pregnancy; (6) blood pressure measured during pregnancy; (7) received iron syrup or tablets during pregnancy; (8) urine sample taken during pregnancy and results discussed; (9) blood sample taken during pregnancy and results discussed; (10) physician-assisted delivery; (11) child delivered in hospital; (12) child delivered in health center; (13) first prenatal care visit within 7 days post-birth; (14) child born low birthweight; (15) child received recommended immunizations; (16) child’s vaccination record cited; (17) child received vitamin A; (18) child received received vitamin K; (19) mother received vitamin A; (20) no breastfeeding at 6 m
Zeng et al.^41^	Quasi-experimental with geographic control	World Bank	Republic of Congo	Enumeration zones reflecting census regions (Niari, Plateaux, Pool, Buenza, and Cuvette)	Health records for 678 pregnant people	Systems-level: performance-based financing	Two geographically proximal regions not implementing an RBF program	(1) Institutional delivery; (2) 1+ antenatal care visit; (3) 3+ antenatal care visit; (4) 1+ postnatal care visit; (5) use of family planning methods; (6) offered and received HIV test; (7) received medications; (8) quality of healthcare; (9) had a bed net; (10) sought curative care; (11) children received BCG and DTP; (12) number of curative visits; (13) number of patients referred to hospital; (14) children fully immunized; (15) children received vitamin A; (16) pregnant women tested for HIV; (17) attended birth; (18) receipt of 2+ tetanus toxoid vaccines

### Risk of bias

Among the 17 randomized clinical trials, eight were deemed at low risk of bias, eight had some concerns, and one was deemed at high risk of bias (Table 2). Among the 15 non-randomized studies, one was deemed to be at low risk of bias, five were at moderate risk of bias, eight were at serious risk of bias, and one was at critical risk of bias (Table 3). The study deemed to be at critical risk of bias was due to self-selection into the intervention and limited comparability between groups^13^. This study was excluded from meta-analyses.

**Table 2:** Risk of bias in randomized controlled trials reporting on interventions to increase the uptake of vaccines recommended during pregnancy

Study author and year	Randomization process	Deviations from the intended intervention(s)	Missing outcome data	Measurement of the outcome	Selection of the reported result	Overall
Patient-level Interventions						
Frew et al.^14^	Some concerns	Low risk	Low risk	Low risk	Low risk	Some concerns
Goodman et al.^15^	Low risk	Low risk	Low risk	Low risk	Low risk	Low risk
Kriss et al.^24^	Some concerns	Low risk	Low risk	Low risk	Low risk	Some concerns
Meharry et al.^17^	Some concerns	Some concerns	Low risk	Low risk	Low risk	Some concerns
Moniz et al.^18^	Low risk	Low risk	Low risk	Low risk	Low risk	Low risk
O’Leary et al.^26^	Low risk	Low risk	Low risk	Some concerns	Low risk	Some concerns
Payakachat et al.^25^	Low risk	Low risk	Low risk	Low risk	Low risk	Low risk
Regan et al.^20^	Low risk	Low risk	Low risk	Low risk	Low risk	Low risk
Sato and Fintan^28^	Low risk	Low risk	Low risk	Low risk	Low risk	Low risk
Stockwell et al.^21^	Some concerns	Low risk	Low risk	Low risk	Low risk	Some concerns
Wong et al.^22^	Some concerns	Low risk	Low risk	Low risk	Low risk	Some concerns
Yudin et al.^23^	Low risk	Low risk	Low risk	Low risk	Low risk	Low risk
Provider or systems-level interventions						
Mwase et al.^37^	Some concerns	Low risk	Low risk	Low risk	Low risk	Some concerns
Wootton et al.^48^	Low risk	Low risk	Low risk	Low risk	Low risk	Low risk
Patient and provider or systems-level interventions						
Chamberlain et al.^45^	Some concerns	Some concerns	Low risk	Low risk	Low risk	Some concerns
Jordan et al.^16^	Low risk	Low risk	High risk	Low risk	Some concerns	High risk
Omer et al.^46^	Low risk	Low risk	Low risk	Low risk	Low risk	Low risk

**Table 3:** Risk of bias in non-randomized reporting on interventions to increase the uptake of vaccines recommended during pregnancy

Study author and year	Confounding	Selection of participants	Classification of interventions	Deviations from intended intervention	Missing Data	Measurement of Outcomes	Selection of Reported Result	Overall
Patient-level interventions								
Chakrabarti et al.^27^	Moderate risk	Low risk	Low risk	Low risk	No information	Low risk	Low risk	Moderate risk
Momani et al.^13^		Low risk	Low risk	No information	No information	Low risk	No risk	Critical risk
Shah et al.^38^	Serious risk	Low risk	Low risk	No information	No information	Low risk	No risk	Serious risk
Provider or systems-level interventions								
Balakrishnan et al.^35^	Serious risk	Low risk	Low risk	No information	No information	No information	Low risk	Serious risk
Bonfrer et al.^36^	Moderate risk	Low risk	Low risk	Low risk	No information	Low risk	Low risk	Moderate risk
Klatt & Hopp^31^	Serious risk	Low risk	Low risk	Low risk	No information	Low risk	Low risk	Serious risk
Li et al.^33^	Moderate risk	Low risk	Low risk	Low risk	Low risk	Serious risk	Low risk	Serious risk
Mouzoon et al.^32^	Serious risk	Low risk	Low risk	No information	No information	No information	Low risk	Serious risk
Spina et al.^34^	Serious risk	Low risk	Low risk	Low risk	Moderate risk	Low risk	Low risk	Serious risk
Woolley et al.^39^	Moderate risk	Low risk	Low risk	No information	No information	Low risk	Low risk	Moderate risk
Patient and provider or systems-level interventions								
Brewer et al.^44^	Moderate risk	Low risk	Low risk	Low risk	No information	Low risk	Low risk	Moderate risk
Dehlinger et al.^42^	Serious risk	Low risk	Low risk	Low risk	No information	Low risk	Low risk	Serious risk
Mazzoni et al.^43^	Moderate risk	Low risk	Low risk	No information	No information	Low risk	Low risk	Moderate risk
Ohly et al. 2018	Serious risk	Moderate risk	Low risk	Low risk	No information	Low risk	Low risk	Serious risk

### Patient-level interventions

Many of the interventions aligned well with evidence-based strategies recommended by the Community Preventive Services Task Force^12^. We identified 18 patient-level, demand-side only studies; three studies investigated the use of patient reminder and recall systems^16,20,21^, nine investigated the use of clinic-based patient education^15–18,22,23,25,26,46^, two studies investigated the use of patient or family incentive rewards through conditional cash transfers^27,28^, two studies investigated the use of persuasive messaging with patient education^14,24^, and one study investigated the use of patient-held immunization records^38^.

Handheld immunization records appeared to increase vaccination rates the most, albeit with low certainty (Table 4), with effect estimates for increasing tetanus toxoid vaccine uptake ranging from 4.85 (95% CI: 3.79, 7.42) to 6.41 (95% CI: 5.36, 7.67). The pooled RR was 5.65 (95% CI: 4.30, 7.42), with possibly substantial heterogeneity (*I*^2^ = 69%, tau = 0.03, *P* = 0.07). These estimates were also derived from multiple sites within a single study, making it difficult to draw firm conclusions. Two studies examined the use of patient incentives via conditional cash transfer programs, where participants received cash stipends in exchange for receiving antenatal care services^27,28^. In Nigeria, Sato and Fintan (2019) showed that payments in the amount of C300 or C800 (equivalent to $2.00 and $5.33 US dollars, respectively) increased tetanus toxoid vaccination modestly (RR 1.38; 95% CI: 1.28, 1.49 and RR 1.56; 95% CI: 1.46, 1.67, respectively)^28^. However, a similar conditional cash transfer program in rural India did not observe a significant effect on tetanus toxoid vaccination (RR 1.04; 95% CI: 0.97, 1.12)^27^. The pooled RR indicated a possible modest effect of conditional cash transfer programs (pooled RR 1.31; 95% CI: 1.03, 1.66) but with possibly considerable heterogeneity (*I*^2^ = 97%; tau=0.04; *P* < 0.001) and low certainty (Table 4).

**Table 4:** Results of grading of recommendations, assessment, development, and evaluations (GRADE) review of interventions to increase vaccine uptake during pregnancy

Intervention	No. of studies	Certainty	Risk of bias	Im-precision	In-consistency	Indirect-ness	Publication bias	Plain language summary
Patient-level interventions	18	Low	Moderate	Low	Moderate	Low	Moderate	Patient-level interventions may have little or no effect on vaccine uptake compared to standard care
Patient education	9	Low	Moderate	Low	Moderate	Moderate	Moderate	Patient education may have little or no effect on vaccine uptake compared to standard care
Patient or family incentive rewards	2	Low	Moderate	Moderate	Moderate	Low	Moderate	Patient or family incentives may have little or no effect on vaccine uptake compared to standard care
Persuasive messaging	2	Very low	Moderate	Very low	Moderate	Low	Moderate	Whether persuasive messaging increases vaccine uptake compared with standard care is very uncertain
Patient reminders/recall	3	Very Low	High	Very Low	High	Low	Moderate	Whether patient reminders increase vaccine uptake compared to standard care is very uncertain.
Patient-held immunization records	1	Low	Low	Moderate	Moderate	Low	Moderate	Patient-held immunization records may have little or no effect on vaccine uptake compared to standard care
Provider or systems-level interventions	11	Very Low	Moderate	Low	Very Low	Low	Moderate	Whether provider or systems-level interventions increase vaccine uptake compared to standard care is very uncertain
AFIX program	3	Very Low	Moderate	Low	Very Low	Low	Moderate	Whether Assessment, Feedback, Incentive, and eXchange programs increase vaccine uptake compared to standard care is very uncertain
Vaccine delivery systems	2	Very Low	Low	Low	Very Low	Low	Moderate	Whether variations to vaccine delivery systems increase vaccine uptake compared to standard care is very uncertain
Performance-based financing	2	Very Low	Moderate	Very Low	Low	Low	Moderate	Whether performance-based financing increases vaccine uptake compared to standard care is very uncertain
Provider reminders	1	Very low	Very low	Low	Very Low	Very Low	Low	Whether provider reminders increase vaccine uptake compared to standard care is very uncertain
Mobile health platforms	1	Very low	Very low	Moderate	Low	Very Low	Low	Whether mobile health platforms increase vaccine uptake compared to standard care is very uncertain
Socially accountable medical education	1	Very Low	Low	Very Low	Very Low	Very Low	Low	Whether socially accountable medical education increases vaccine uptake compared to standard care is very uncertain
Patient and provider or systems-level interventions	6	Very Low	Moderate	Low	Very Low	Low	Moderate	Whether patient and provider or systems-level interventions increase vaccine uptake compared to standard care is very uncertain

**Fig. 2:**
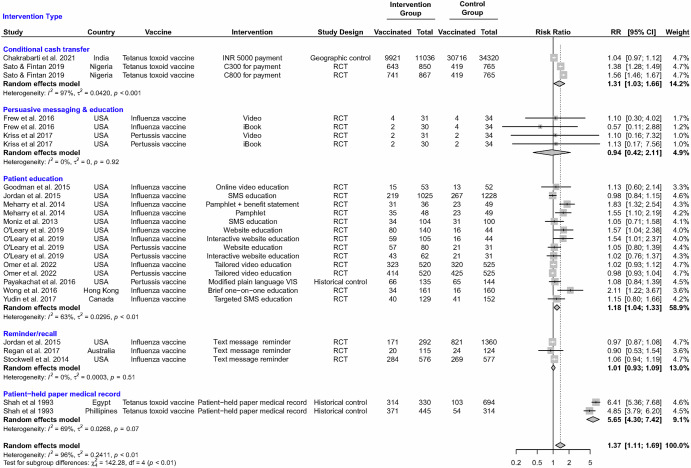
Effect of patient-level (demand-side) interventions. Individual and pooled effect estimates for interventions that target patient-level demand-side interventions to increase the uptake of recommended vaccines during pregnancy. Pooled effect estimates were estimated by random effects meta-analysis overall and by intervention. CI indicates 95% confidence intervals, and RR indicates relative risk estimates.

Other patient-level interventions showed more modest effects on vaccine uptake during pregnancy. Although most studies evaluated the use of patient education strategies, these showed little to moderate improvements in vaccination rates and were entirely implemented in HIC settings. Patient education studies used printed materials (i.e., posters, statements, or pamphlets) or electronic materials (i.e., interactive website, videos, or text messages). One study used brief one-on-one education with a healthcare provider^22^. Effect estimates ranged from 0.98 (95% CI: 0.84, 1.15)^16^ to 2.11 (95% CI: 1.22, 3.67)^22^, with three studies showing a significant improvement in influenza and/or pertussis vaccine uptake^17,22,26^. The pooled RR indicated a modest effect of patient education on influenza or pertussis vaccine uptake (pooled RR 1.18; 95% CI: 1.04, 1.33), with possibly substantial heterogeneity (*I*^*2*^ = 63%; tau = 0.03; *P* < 0.01) (Fig. 2) and low certainty (Table 4). Results were similar when we considered any study implementing the effect of patient education, either alone or in combination with other interventions, and pooled RRs by vaccine suggested a possible modest increase in influenza vaccine uptake (pooled RR 1.21; 95% CI: 1.06, 1.37) but no increase in pertussis vaccine uptake (pooled RR 1.00; 95% CI: 0.96, 1.04) (Fig. S2).

Three studies evaluated the use of text message-based immunization reminders. Effect estimates for the use of text message reminders ranged from 0.90 (95% CI: 0.53, 1.54)^20^ to 1.06 (95% CI: 0.94, 1.19)^21^. The pooled RR indicated no effect of SMS-based immunization reminder systems on influenza vaccine uptake (pooled RR 1.01; 95% CI: 0.93, 1.09), with possibly low heterogeneity (*I*^*2*^ < 0.1; tau < 0.01; *P* = 0.51) and very low certainty (Table 4). Two small US studies (*n* < 36 per group) evaluated the use of different persuasive messaging using an affective messaging video or iBook^14,24^. Neither study showed a significant effect on influenza or pertussis vaccine uptake (pooled RR 0.94; 95% CI: 0.42, 2.11; *I*^2^ < 0.1; tau < 0.01; *P* = 0.92), and the evidence in support of a persuasive message was deemed to have very low certainty (Table 4).

Across all studies evaluating patient-level interventions exclusively, the evidence suggested a possible modest effect of patient-level interventions (pooled RR 1.37; 95% CI: 1.11, 1.69) with possibly considerable heterogeneity (*I*^2^ = 96%; tau = 0.24; *P* < 0.001) (Fig. 2). Among the 12 randomized controlled trials, six were deemed to be at low risk of bias, and six had some concerns (Table 2). Among the three quasi-experimental studies, one was at moderate risk, one at serious risk, and one at critical risk of bias (Table 3). Results from the six randomized controlled trials at low risk of bias showed no effect of patient education (*n* = 5/5)^15,18,20,23,25^ but increases in tetanus vaccination after cash incentives (*n* = 1)^28^.

We identified little evidence for publication bias (Egger statistic = 1.46; *P* = 0.33). However, examination of funnel plots showed more than expected larger studies with positive findings identified in the literature for patient-level interventions (Fig. S3), including patient education interventions (Fig. S4). Patient-level evaluations were deemed to have low certainty evidence in increasing vaccine uptake during pregnancy (Table 4).

### Systems or provider-level interventions

Among the 11 studies of supply-side only interventions, three studies trialed some form of an Assessment-Feedback-Incentives-eXchange (AFIX) quality improvement program to increase influenza (*n* = 1) or both influenza and pertussis (*n* = 2) vaccine uptake^32,34,46^, three trialed the use of performance-based financing to improve antenatal care services (including tetanus toxoid vaccination)^36,37,40^, two trialed vaccine delivery systems for influenza or pertussis vaccination^33,48^, one trialed the use of a provider reminder system^31^, one trialed the use of a mobile health platform for improving the continuum of antenatal care^35^, and one trialed the use of socially-accountable medical education^39^.

**Fig. 3:**
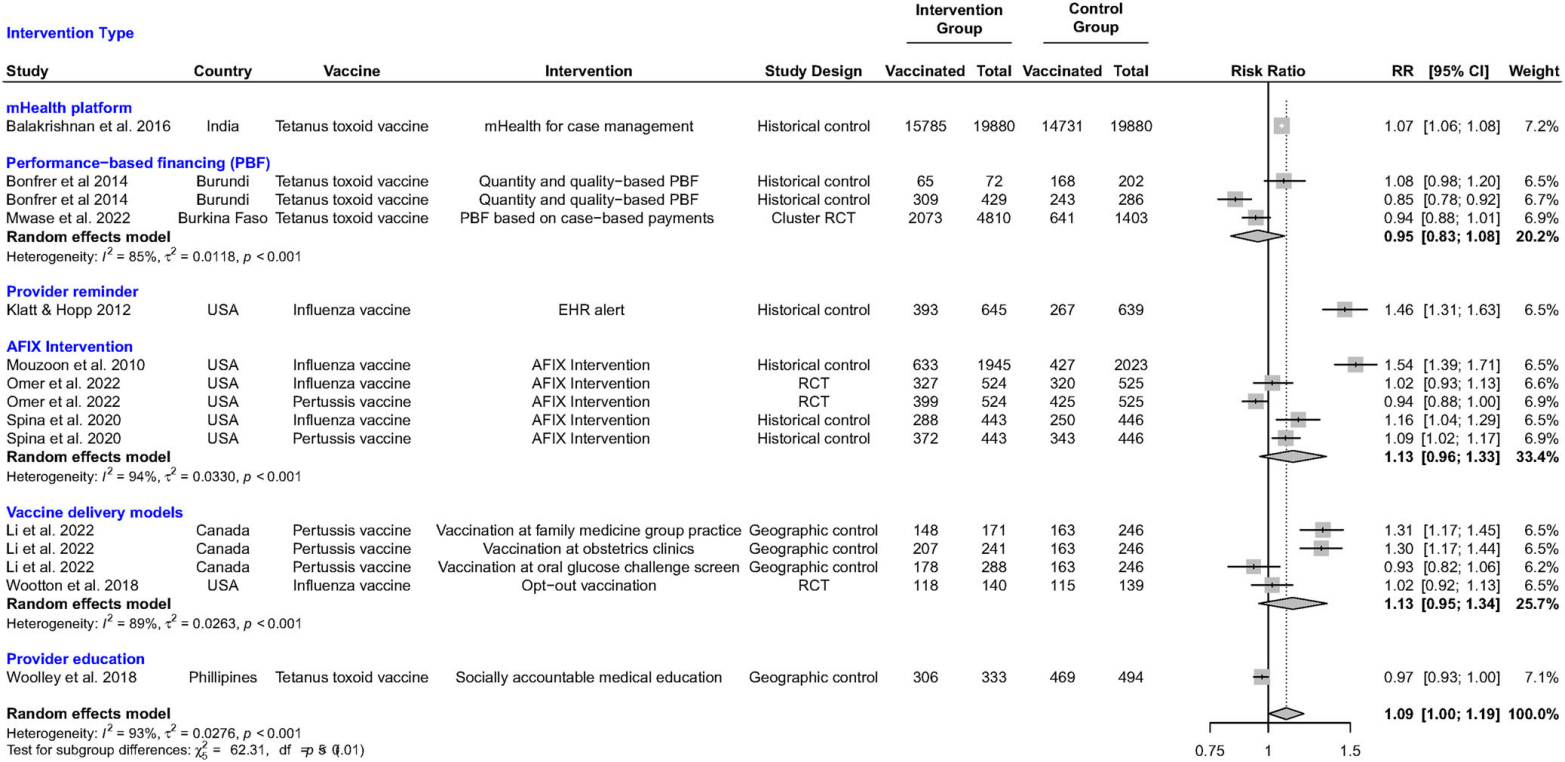
Effect of supply-side interventions targeting health systems and/or healthcare providers. Individual and pooled effect estimates for interventions that target health systems and/or healthcare providers to increase the uptake of recommended vaccines during pregnancy. Pooled effect estimates were estimated by random effects meta-analysis overall and by intervention. AFIX indicates Assessment, Feedback, Incentive, eXchange programs, CI indicates 95% confidence intervals, and RR indicates relative risk estimates.

Results did not strongly support supply-side interventions that were evaluated. Among AFIX interventions, effect estimates ranged from 0.94 (95% CI: 0.88, 1.00)^46^ to 1.54 (95% CI: 1.39, 1.71)^32^, with a pooled RR estimate of 1.13 (95% CI: 0.96, 1.33) with possibly considerable heterogeneity (*I*^2^ = 94%, tau = 0.03; *P* < 0.001) (Fig. 3) and very low certainty (Table 4). Effect estimates from the two LMIC-based studies of performance-based financing to increase tetanus toxoid vaccination ranged from 0.85 (95% CI: 0.78, 0.92)^36^ to 1.08 (95% CI: 0.98, 1.20)^36^. The pooled RR was 0.95 (95% CI: 0.83, 1.08), with possibly considerable heterogeneity (*I*^2^ = 85%; *P* < 0.001) and very low certainty (Table 4). The single study in India that evaluated the use of a mobile health (mHealth) platform for supporting case management and continuum of care for maternal and child health services showed a modest improvement in tetanus toxoid vaccination (RR 1.07; 95% CI: 1.06, 1.08)^35^ with very low certainty. The US study that evaluated the use of a healthcare provider reminder via an electronic health record alert, showing a 46% improvement in influenza vaccination rates (RR 1.46; 95% CI: 1.31, 1.63)^31^ with very low certainty. A study in Canada trialed different vaccine delivery systems and showed that administration of pertussis vaccine in obstetrics clinics or family medicine group practices resulted in higher uptake compared to the pre-intervention standard of vaccine administration in community clinics (RR 1.30; 95% CI: 1.17, 1.44 and RR 1.31; 95% CI: 1.17, 1.45, respectively)^33^ with very low certainty. Finally, one study in the Philippines investigated the use of a socially accountable medical education program to improve community health services, which admitted students who more accurately reflected the geographical, ethnic, and socio-economic diversity of the school’s reference population and applied an extended community-engaged service learning model with locally relevant curriculum. The socially accountable medical education program showed no effect on tetanus toxoid vaccination (RR 0.97; 95% CI: 0.93, 1.00)^39^ with very low certainty.

Results were similar when we considered provider or systems-level interventions when administered alone or in combination with other interventions. Seven studies evaluated provider education, either alone or in combination with patient-level interventions (Fig. S5). Pooled RR showed a modest improvement in influenza vaccine uptake (pooled RR 1.20; 95% CI: 1.02; 1.42) with possibly considerable heterogeneity (*I*^2^ = 89%; *P* < 0.001). A modest effect was similarly observed for pertussis vaccine (pooled RR 1.63; 95% CI: 0.74, 3.57), but with wider confidence intervals and possibly considerable heterogeneity (*I*^2^ = 92%; *P* < 0.001). Three studies examined provider reminders, either alone or in combination with other interventions (Fig. S6), and these showed modest improvement in either tetanus toxoid or influenza vaccine uptake (pooled RR 1.18; 95% CI: 0.97, 1.44) with possibly considerable heterogeneity (*I*^2^ = 94%; *P* < 0.001). Six US studies evaluated the nomination of an immunization champion (i.e., a dedicated member of staff who was compensated for championing immunization services and implementing quality improvement interventions) and enhanced electronic immunization documentation within practices (Fig. S7), showing possible modest improvement in influenza vaccine uptake (pooled RR 1.22; 95% CI: 1.04, 1.43) and pertussis vaccine uptake (pooled RR 1.39; 95% CI: 1.03, 1.57). However, meta-analyses indicated possibly considerable heterogeneity for both (*I*^2^ = 87% and *I*^2^ = 91%, respectively). Five studies evaluated the use of standing orders and AFIX programs and showed possible modest improvements in influenza vaccination (pooled RR 1.21; 95% CI: 1.03, 1.43) and pertussis vaccination (pooled RR 1.37; 95% CI: 0.99, 2.47), both with possibly considerable heterogeneity (*I*^2^ = 90% and *I*^2^ = 92%, respectively) (Fig. S8).

Across all studies evaluating provider or systems-level interventions exclusively, the evidence suggested a possible small effect of provider or systems-level interventions (pooled RR 1.09; 95% CI: 1.00, 1.19) with possibly considerable heterogeneity (*I*^2^ = 93%; tau=0.03; *P* < 0.001) (Fig. 3). Among the two randomized controlled trials, one was deemed to be at low risk of bias, and one had some concerns (Table 2). Among the seven quasi-experimental studies, two were at moderate risk, and five were at serious risk of bias (Table 3). Results from the one randomized controlled trial at low risk of bias found no increase in influenza vaccination during pregnancy after an opt-out approach to immunization^48^.

We observed no statistical evidence of publication bias in systems or provider-level interventions (Egger statistic = 0.28; *P* = 0.83); however, examination of funnel plots showed more studies with positive findings than would be expected appear in the published literature for provider or systems-level interventions (Fig. S9), including provider education (Fig. S10), provider reminders (Fig. S11), immunization champions and enhanced vaccine documentation (Fig. S12), and standing orders and AFIX programs (Fig. S13).

### Patient and provider or systems-level interventions

**Fig. 4:**
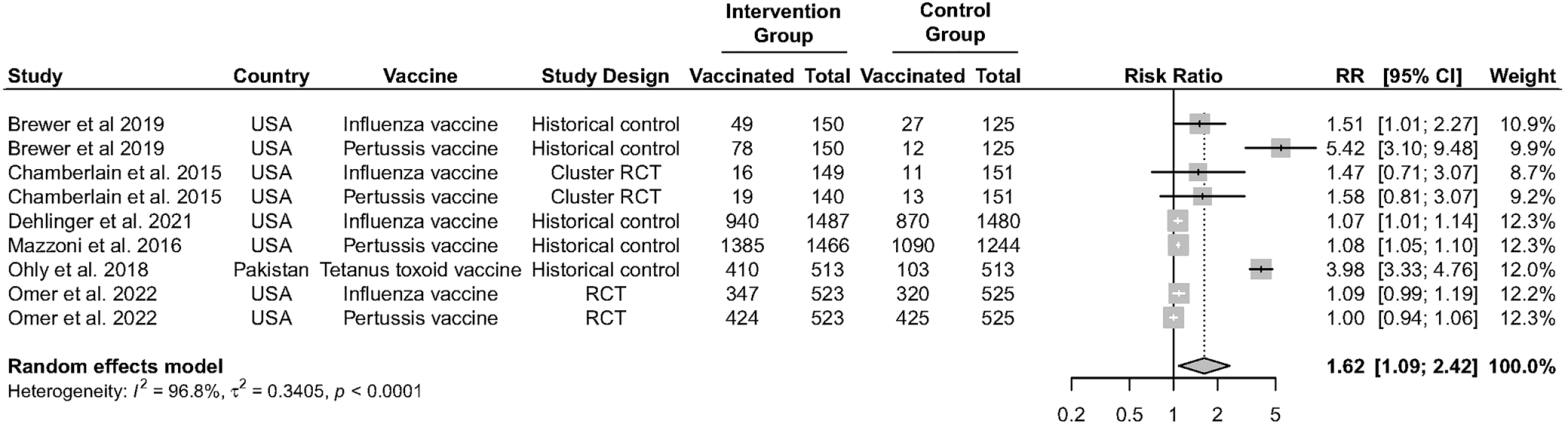
Effect of demand and supply-side interventions. Individual and pooled effect estimates for demand and supply-side interventions to increase the uptake of recommended vaccines during pregnancy. Pooled effect estimates were estimated by random effects meta-analysis. CI indicates 95% confidence intervals, and RR indicates relative risk estimates.

Six studies were identified that evaluated ‘bundled’ multi-level interventions that addressed both demand and supply-side factors (Fig. 4)^42–47^. Five of these studies were conducted in the US and one in Pakistan. Bundled interventions often incorporated patient-level components like patient education and provider and systems-level components such as AFIX, enhanced vaccine documentation, and standing orders for vaccination. Effect estimates ranged from 1.00 (95% CI: 0.94, 1.06)^36^ to 5.42 (95% CI: 3.10, 9.48)^34^. Among the two randomized trials in the US, neither showed a significant effect of multi-level interventions on influenza or pertussis vaccine uptake^45,46^. Three US studies using a historical control reported a significant increase in influenza vaccine uptake^42^, pertussis vaccine uptake^43^, or influenza and pertussis vaccine uptake^44^. The pooled RR for multi-level interventions was 1.62 (95% CI: 1.09, 2.42) with possible high heterogeneity (*I*^2^ = 97%; *P* < 0.001) (Fig. 4) and very low certainty (Table 4). Among the three randomized controlled trials, one was deemed to be at low risk of bias, one had some concerns, and one was at high risk of bias (Table 2). Among the four quasi-experimental studies, two were at moderate risk of bias, and two were at serious risk of bias (Table 3). Results from the one randomized controlled trial at low risk of bias found no increase in influenza or pertussis vaccination during pregnancy after a multi-level intervention^46^. We observed no statistical evidence of publication bias (Egger statistic = 3.69; *P*-value = 0.14); however, the funnel plot indicated some asymmetry in studies with more positive findings published (Fig. S14).

### Ongoing studies

We identified seven ongoing registered clinical trials describing interventions to increase vaccination rates in pregnant people (Table S2). Four studies are being conducted in the US, two in Italy, and one in the Netherlands. Two target COVID-19 vaccine uptake, two target both influenza and pertussis vaccine uptake, one targets pertussis vaccine uptake exclusively, and another influenza vaccine uptake exclusively, and one targets respiratory syncytial virus vaccine uptake. Interventions focused on patient education, the AFIX framework, motivational interviewing, or tailored decision aids.

## Discussion

This systematic review and meta-analysis synthesizes the existing evidence from completed randomized controlled trials and quasi-experimental studies of interventions to increase vaccine uptake during pregnancy and provides a summary of ongoing studies in this field. Interventions that showed modest improvement in vaccine uptake included patient incentives, patient-held immunization records, provider reminders, and multi-level interventions that address patient, provider, and systems-level factors. However, the number of studies for individual interventions were small, there was considerable heterogeneity in the results of included studies, and with exception to patient incentives, studies deemed to be at low risk of bias did not report significant effects of patient, provider, or patient and provider-level interventions on vaccine uptake during pregnancy. As a result, the evidence from the existing literature provides low to very low certainty in the effects of these interventions. Further research on interventions to increase vaccine uptake during pregnancy is needed.

Several studies supported the effectiveness of demand-side patient-level interventions, but with low certainty. Patient incentives appeared to increase vaccination rates, although only in one of two studies and only in an LMIC context. Although these findings align with the Community Preventive Services Task Force recommendations for strategies to increase vaccination uptake^12^, there were several notable differences. For example, patient education was the most commonly evaluated demand-side intervention identified in this review, yet we found that seven of the nine studies reported no increase in vaccine uptake during pregnancy associated with patient education^15,16,18,23,25,26,46^. Furthermore, given the evidence that studies with positive findings were over-represented in the literature, any benefits of patient-level interventions, including patient education, could be overstated. While evidence supports patient education as an intervention to improve vaccine uptake^12^, prior studies have mostly targeted non-pregnant populations, and it is possible that patient education is less effective for pregnant people. Similarly, although evidence strongly supports reminder/recall strategies to increase vaccination rates^12^, we observed consistent evidence against any significant effect of reminder/recall interventions on vaccination rates in pregnant people. Overall, these findings indicate that although these strategies may be effective in other patient groups, when administered on their own or bundled with other strategies, they may be less effective in increasing vaccination rates in pregnant people.

Supply-side provider or systems-level interventions were less commonly effective in increasing vaccine uptake, with very low certainty in the evidence in support of the effectiveness of provider or systems-level interventions. Although few studies focused on provider reminders, one study showed modest improvement in vaccination rates following a provider reminder system^31^. Results for other provider or systems-level interventions were more mixed, and evidence suggesting possible publication bias further reduces certainty in the effects of these types of interventions. Additional studies investigating the effects of provider or systems-level interventions are needed to understand the effects of such interventions with more certainty.

Three of the four studies examining the effect of multi-level bundled interventions that incorporated AFIX, standing orders, immunization champions, and enhanced immunization documentation showed an indication of modest improvement in influenza and pertussis vaccine uptake during pregnancy. However, these were exclusively implemented in one or two US states, meta-analyses showed considerable heterogeneity, and the evidence was deemed to be of very low certainty. Further, because these interventions were often bundled, it is difficult to identify which components effectively increased vaccination uptake. Additional well-designed randomized controlled trials should investigate these strategies. Studies that can isolate and report on the effects of components within bundled interventions would also be informative.

There were several recommended interventions by the Community Preventive Services Task Force that were not identified in the published literature. No study examined community-wide education to improve vaccination rates in pregnancy^12^, although several ongoing, unpublished studies have been planned to evaluate community education programs. We identified no studies that evaluated the effectiveness of interventions to reduce patient out-of-pocket vaccination costs, administering maternal vaccines in government support programs like Women, Infants, and Children (WIC) clinics or home visits. One study did evaluate an intervention that enhanced the scheduling of existing home visits and showed a 7% relative improvement in tetanus toxoid vaccination^35^, although home visits were the norm in the study setting and available across treatment groups. These may present additional opportunities to explore to increase vaccination rates during pregnancy.

We noted several important differences in the interventions and study designs implemented in HIC vs LMIC settings. Studies in LMICs often utilized systems-level interventions and targeted uptake of tetanus toxoid vaccine as one of many maternal health care outcomes. In contrast, studies in HICs more commonly focused on vaccination during pregnancy (or the perinatal period) as the primary outcome and most commonly trialed patient-level interventions. We also identified differences in the class of interventions implemented in HIC vs LMIC settings, with three LMIC studies compared with 13 in HIC settings. Patient-level interventions evaluated in LMIC studies included the use of patient incentives (via conditional cash transfers) and patient-held immunization records, which were not investigated in HIC studies. Studies in LMIC settings often evaluated systems-level interventions that aimed to improve the continuum of antenatal care or monetarily incentivize health care system improvements. These interventions were also not identified in any of the HIC studies. In HIC studies, AFIX programs and vaccine delivery systems were more commonly implemented. Six HIC studies investigated the use of AFIX as part of a multi-level, bundled intervention, and just one such study was conducted in an LMIC setting. The differences in interventions investigated are likely due to the unique health care systems, infrastructure, and patient populations across countries. However, failure to examine the effect of different interventions in settings could present missed opportunities to identify effective interventions. No HIC or LMIC study involved community input in the design of the intervention, and future evaluations of additional interventions with community input could ensure interventions are culturally appropriate and offer better success.

This review had several strengths and limitations. Despite the use of an experienced research librarian and an inclusive, peer-reviewed search strategy applied to multiple databases, we failed to identify 11 studies that were only retrievable through reference lists and clinical trial registries. This indicates that screening of additional resources is important for comprehensively identifying all relevant studies on this subject. Furthermore, over half of the studies included in this review were conducted in the United States, and no study evaluated the use of AFIX principles outside of the United States. Additional studies outside of the United States would be useful for understanding the effect of these interventions globally. Finally, we identified substantial heterogeneity when pooling most of the effect estimates from studies. While subgroup analyses that separated interventions by vaccine target (i.e., reducing outcome heterogeneity) reduced some heterogeneity, this did not resolve the issue entirely. It is possible that differences in patient populations, healthcare settings, and intervention components could additionally contribute to statistical heterogeneity; however, the limited number of studies addressing each intervention makes evaluating additional sources of heterogeneity challenging. As additional studies are published, further consideration for these sources of heterogeneity would be useful.

With a RSV vaccine recently introduced for pregnant people and the impending introduction of GBS vaccines, the suboptimal vaccination rates in pregnant people are a growing public health concern. The increasing number of vaccines recommended during pregnancy introduces additional challenges for implementation and may result in increasing maternal vaccine hesitancy. Recent data in the US indicate the problem may be worsening, with declining rates of some vaccines and increasing rates of vaccine hesitancy during pregnancy^49,50^. Effective strategies to increase vaccination uptake in this priority population are needed to actualize the benefits of maternal vaccination programs and ensure the success of future maternal vaccine programs. While our findings point toward several promising strategies, further research is needed to fully understand the benefits of these strategies in addition to strategies not yet investigated. Evidence-based strategies will be needed to increase and maintain high levels of immunization during pregnancy, and such evidence is needed for programs in both HIC and LMIC settings.

## Methods

We conducted a systematic review and meta-analysis of the published literature. The study protocol, including the review question, search strategy, information on the relevant participant, intervention, comparison, and outcome (PICO) criteria for inclusion, types of studies to be included, data elements for extraction and data extraction procedures, risk of bias assessment, and strategies for data synthesis and meta-analysis, was prospectively registered (Prospero #CRD42023441073). We included all randomized controlled trials and quasi-experimental studies that measured the effect of an intervention on the uptake of at least one recommended vaccine during pregnancy. Our primary outcome of interest was uptake of recommended vaccines during pregnancy, as measured by the percent of pregnant participants who received a recommended vaccine. Interventions may target pregnant people directly or target healthcare systems or healthcare providers. Eligible studies included either a standard care comparator or an active comparison group. Because our research question aims to measure the effects of interventions on health care, and randomized trials were feasible to address our research question, in line with Cochrane guidance^51^, our focus was on randomized trials, including parallel arm, stepped wedge, and cluster randomized trials. We additionally included quasi-experimental design studies (e.g., pre/post, geographic controls), since some systems-level or policy interventions may be difficult to evaluate using a randomized trial. This systematic review therefore excluded non-experimental, observational studies of interventions, review articles, commentaries, and letters to the editor. We did not restrict studies based on language and used online translation services (e.g., translate.google.com) and library services to translate articles not published in English. We did not place any restrictions on the follow-up period in the included studies. We excluded studies that (1) were not a primary study (e.g., conference abstract, review, commentary); (2) did not use an experimental or quasi-experimental study design; (3) did not address vaccine uptake, intent, or hesitancy/acceptance of a recommended vaccine during pregnancy; or (4) did not evaluate the effect of an intervention.

The electronic search strategy was developed in collaboration with a Cochrane Gynecological, Neuro-oncology and Orphan Cancers information specialist (JP, see acknowledgements) and was peer-reviewed by a second independent information specialist (BS, see acknowledgements). Published literature was searched using Cochrane Central Register of Controlled Trials (CENTRAL), MEDLINE Ovid, Embase Ovid, and CINAHL EBSCO (Cumulative Index to Nursing and Allied Health Literature). The complete search strategy for each database is included in the Web Supplement (Supplementary Note A–E). In addition, we identified ongoing and unpublished trials using the ISRCTN registry (www.isrctn.com), US National Institutes of Health Ongoing Trials Register ClinicalTrials.gov (www.clinicaltrials.gov), *World Health Organization International Clinical Trials Registry Platform (ICTRP) Search Portal* (apps.who.int/trialsearch), Australian New Zealand Clinical Trials Registry (www.anzctr.org.au), EU Clinical Trials Register (www.clinicaltrialsregister.eu). Reference lists of all included studies and relevant systematic reviews were screened to identify any further relevant studies.

Two reviewers screened titles and abstracts of identified studies to assess eligibility for inclusion. Articles deemed eligible for inclusion based on title and abstract screening were selected for full text review; a random 10% of excluded articles were reviewed and verified by an independent author to ensure accuracy of exclusions. A review of full-text articles was performed by two review authors independently. Disagreements between reviewers were resolved by a third review author. The selection process adhered to PRISMA guidelines for high-quality systematic reviews (Supplementary Note F)^52^. The research team aims to update the search every five years and update results accordingly, with the next anticipated update in July 2028.

### Data extraction

Data extraction was performed using a pre-tested, standardized data extraction form following pilot testing on the first 10% of included citations. Two reviewers independently extracted data (H.U., E.O., S.R.). Disagreements were resolved by a third independent reviewer (A.K.R.). Extracted data elements from each eligible citation included the study design, date and duration of study, number of study centers or locations, study setting, number of participants/clusters randomized, baseline differences between treatment groups, description of intervention(s) and comparison group(s), target of the intervention (i.e., healthcare system, healthcare providers, or pregnant patients), primary and secondary outcomes measured, method of outcome measurement, types of statistical analyses performed, counts of vaccinated participants in the intervention and control group, and relative risks and 95% confidence intervals (where provided).

**Fig. 5:**
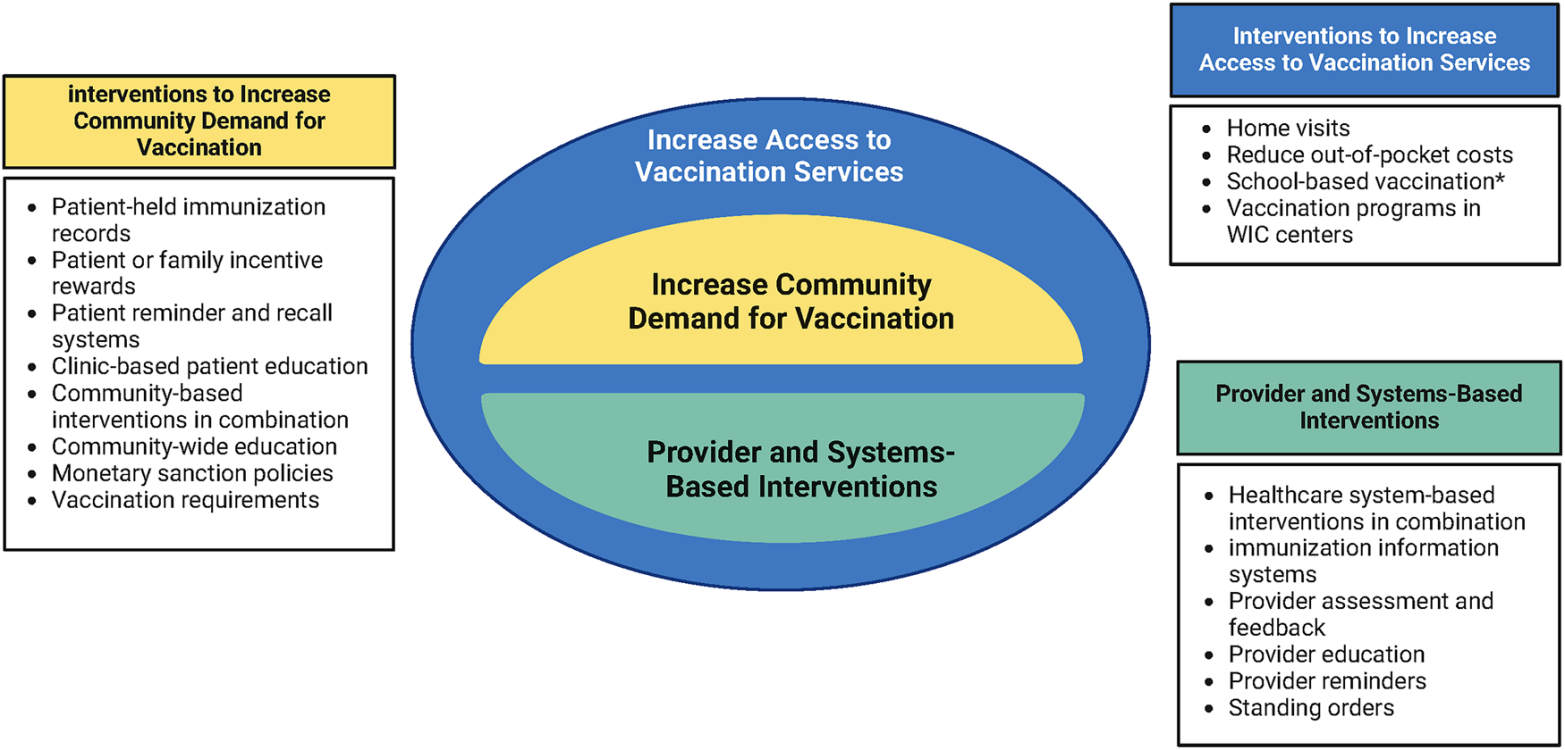
Community preventive services task force framework for increasing vaccination. The Community Preventive Services Task Force framework for increasing vaccination rates recommends using a combination of community-based and healthcare system-based interventions to increase demand and supply of vaccines (SOURCE: https://www.thecommunityguide.org/topics/vaccination.html).

We applied the framework developed by the Community Preventive Services Task Force for increasing vaccination^12^ to identify interventions represented in the literature, which includes: (1) those focused on increasing patient demand; (2) those directed at health care providers; and (3) those that enhance access to vaccinations (Fig. 5). Based on this, interventions were classified into patient-level (demand-side interventions), provider or systems-level (supply-side interventions), or a combination of patient, provider, and systems levels (demand- and supply-side interventions).

### Data synthesis

We considered the intention-to-treat effects of interventions and conducted a separate meta-analysis of treatment effects for demand-side interventions, supply-side interventions, and demand and supply-side interventions. Where provided by the study authors, we used information on relative risk to inform meta-analyses. When relative risks were not reported, we manually calculated these using reported counts. We conducted random effects meta-analysis to estimate the treatment effect of study interventions. We conducted subgroup analyses by the intervention type and recommended vaccine, where possible. The *I*^2^ and tau statistics were used to estimate heterogeneity^53^. We considered <30% to be possibly low, 30–60% to be possibly moderate, 50–90% to be possibly substantial, and 75–100% to be possibly considerable^53^.

Risk of bias for randomized and cluster randomized control trials was assessed using version 2 of the Cochrane risk-of-bias (RoB 2) tool^54^. Risk of bias in quasi-experimental studies (non-randomized) was assessed using the ROBINS-I tool^55^. Two investigators (H.U. and S.R.) independently rated each study. A third investigator (A.K.R.) performed a consensus review, and team discussion occurred where there were conflicts. The likelihood of publication bias was assessed using the Egger statistic and by examining symmetry in funnel plots^56,57^.

Finally, we evaluated the certainty of the evidence in support of each intervention type using GRADE criteria^58^. Two investigators (A.K.R., S.R.) independently rated each intervention using GRADE criteria. Where there were conflicts, consensus was reached through consultation with the wider study team.

### Patient and public involvement

This study did not include input or participation from patients or the public in the design, conduct, or interpretation of results.

## Supplementary information

Supplementary information

## Data Availability

The extracted data summarized in this review are available to researchers in an Open Science Framework repository using the following link: https://osf.io/rmxny/?view_only=a81bb3a49669458282d75ee2eccefb65.

## Code availability

R code for conducting meta-analyses with the extracted data is available in an Open Science Framework repository here: https://osf.io/rmxny/?view_only=a81bb3a49669458282d75ee2eccefb65.
