# Supplementary material for: Systematic review and meta-analysis of interventions to increase the uptake of vaccines recommended during pregnancy
Source: NPJ Vaccines. 2025 Apr 19;10:76. doi: 10.1038/s41541-025-01120-1 (PMC12009365; doi:10.1038/s41541-025-01120-1)
Supplement: Supplementary file 1 — Supplementary information [file 41541_2025_1120_MOESM1_ESM.pdf]

Supplement to: Regan AK, Uwimana H, Rowe SL, Olsanska EJ, Agnew B, Castillo E, Fiddian-Green A, Giles ML. Systematic Review & Meta-analysis of Interventions to Increase the Uptake of Vaccines Recommended during Pregnancy.

Table of Contents

Table S1. Summary of full text articles excluded from review with reason for exclusion (n=32)……………...4

Figure S1. Geographic location of studies identified that evaluate interventions to increase vaccine uptake during pregnancy………8

Table S2. Description of interventions to increase vaccine uptake during pregnancy……………………………9

Table S3. Ongoing registered clinical trials aiming to increase the uptake of vaccines recommended during pregnancy………..28

Figure S2. Effect of patient education interventions to increase the uptake of recommended vaccines during pregnancy, by recommended vaccine……………………………………………………………………………32

Figure S3. Funnel plot of patient-level interventions to increase uptake of recommended vaccines during pregnancy……..33

Figure S4. Funnel plot of patient education intervention to increase uptake of recommended vaccines during pregnancy………..34

Figure S5. Effect of provider education interventions to increase the uptake of recommended vaccines during pregnancy, by recommended vaccine……………………………………………………………………………35

Figure S6. Effect of provider reminder interventions to increase the uptake of recommended vaccines during pregnancy, by recommended vaccine……………………………………………………………………………36

Figure S7. Effect of interventions with immunization champion and enhanced vaccine documentation to increase the uptake of recommended vaccines during pregnancy, by recommended vaccine……………..37

Figure S8. Effect of standing orders and Assessment, Feedback, Incentives, and eXchange (AFIX) programs to increase the uptake of recommended vaccines during pregnancy, by recommended vaccine…….38

Figure S9. Funnel plot of systems or provider-level interventions to increase uptake of recommended vaccines during pregnancy……………………………………………………………………………………………………..39

Figure S10. Funnel plot of provider education intervention to increase uptake of recommended vaccines during pregnancy………..40

Figure S11. Funnel plot of provider reminder intervention to increase uptake of recommended vaccines during pregnancy………..41

Figure S12. Funnel plot of interventions with immunization champions and enhanced vaccine documentation to increase the uptake of recommended vaccines during pregnancy……………………………42

Figure S13. Funnel plot of interventions with standing orders and Assessment, Feedback, Incentives, and eXchange (AFIX) programs to increase the uptake of recommended vaccines during pregnancy……..……43

Figure S14. Funnel plot of patient and provider or systems-level interventions to increase vaccine uptake during pregnancy……..44

Supplementary Note A. Central Search Strategy: Interventions to increase vaccine uptake during pregnancy…….45

Supplementary Note B. CINAHL Search Strategy: Interventions to increase vaccine uptake during pregnancy**…….……….**46

Supplementary Note C. Embase Search Strategy: Interventions to increase vaccine uptake during pregnancy………..48

Supplementary Note D. Medline Search Strategy: Interventions to increase vaccine uptake during pregnancy………..50

Supplementary Note E. SCOPUS Search Strategy: Interventions to increase vaccine uptake during pregnancy………..52

Supplementary Note F. PRISMA checklist for systematic reviews and meta-analyses…………………………..54

Table S1. Summary of full text articles excluded from review with reason for exclusion (n=32).

Author & Year	Sourcde	Title	Reason for exclusion
**Binyaruka et al. 2015**	PloS One	Effect of Paying for Performance on Utilisation, Quality, and User Costs of Health Services in Tanzania: a Controlled Before and After Study	Does not measure vaccine uptake, intent or hesitancy/acceptance during pregnancy
**Celep et al. 2020**	Turkish Journal of Medical Sciences	Different perspectives of immunizations during pregnancy	Not a randomized trial or quasi-experimental design
**Chamberlain et al. 2016**	Human Vaccines & Immunotherapeutics	Impact of a multi-component antenatal vaccine promotion package on knowledge, attitudes and beliefs about influenza and Tdap vaccination during pregnnacy	Reported on different outcomes from same study [combined with citation Chamberlain et al. 2015]
**Cheng et al. 2023**	BMC Pregnancy & Childbirth	Using the teach-back method to improve postpartum maternal-infant health among women with limited maternal health literacy: a randomized controlled study	Does not measure vaccine uptake, intent or hesitancy/acceptance during pregnancy
**Coleman et al. 2020**	Reproductive Health	Evaluating the effect of maternal mHealth text messages on uptake of maternal and child health care services in South Africa: a multicentre cohort intervention study	Does not measure vaccine uptake, intent or hesitancy/acceptance during pregnancy
**Dudley et al. 2020**	Vaccine	Factors associated with referring close contacts to an app with individually-tailored vaccine information	Does not measure vaccine uptake, intent or hesitancy/acceptance during pregnancy
**Engineer et al. 2016**	International Journal of Epidemiology	Effectiveness of a pay-for-performance intervention to improve maternal and child health services in Afghanistan: A cluster-randomized trial	Does not measure vaccine uptake, intent or hesitancy/acceptance during pregnancy
**Frew et al. 2022**	Vaccine	Development of effective messages to promote maternal immunization in Kenya	Does not measure vaccine uptake, intent or hesitancy/acceptance during pregnancy

**Furbetta & Paponi 1977**	Revista Italiana d'Igiene	Vaccination during pregnancy	Not a primary study
**Gagneur et al. 2019**	Euro Surveillance	Promoting vaccination in maternity wards – motivational interview technique reduces hesitancy and enhances intention to vaccinate, results from a multicentre non-controlled pre- and post-intervention RCT-nested study, Quebec, March 2014 to February 2015	Does not measure vaccine uptake, intent or hesitancy/acceptance during pregnancy
**Hanon & Ali 2022**	Pakistan Journal of Medical and Health Sciences	Effectiveness of Instructional Program on Women Practices about Prevention of COVID-19 in Kirkuk City	Does not measure vaccine uptake, intent or hesitancy/acceptance during pregnancy
**Hashemi et al. 2017**	Koomesh	An investigation of educational intervention impact on pregnant women for promote preventive behaviors of influenza H1N1: Using health belief model. [Persian]	Does not measure vaccine uptake, intent or hesitancy/acceptance during pregnancy
**Heryani & Lestari 2022**	Indian Journal of Forensic Medicine and Toxicology	Digital Pocketbook increase mother's knowledge about COVID-19 transmission prevention	Does not measure vaccine uptake, intent or hesitancy/acceptance during pregnancy
**Jones et al. 2016***	Infectious Diseases in Obstetrics & Gynecology	Efforts to Improve Immunization Coverage during Pregnancy among Ob-Gyns	Does not measure vaccine uptake, intent or hesitancy/acceptance during pregnancy
**Kapoor et al. 2022**	BMJ Open	Effect of lifting COVID-19 restrictions on utilisation of primary care services in Nepal: A difference-in-differences analysis	Does not measure vaccine uptake, intent or hesitancy/acceptance during pregnancy
**Kitzman et al. 1997**	JAMA: Journal of the American Medical Association	Effect of prenatal and infancy home visitation by nurses on pregnancy outcomes, childhood injuries, and repeated childbearing. A randomized controlled trial	Does not measure vaccine uptake, intent or hesitancy/acceptance during pregnancy
**Lewycka et al. 2010**	Trials	A cluster randomised controlled trial of the community effectiveness of two interventions in	Not a primary study

		rural Malawi to improve health care and to reduce maternal, newborn and infant mortality	
**Malhotra & Kaur 2013**	Annals of Nutrition and Metabolism	Nutrition education intervention improves nutritional status and healthcare seeking practices of pregnant women in low-income settings	Not a primary study
**Marcell et al. 2022**	American Journal of Obstetrics & Gynecology	One Vax Two Lives: a social media campaign and research program to address COVID-19 vaccine hesitancy in pregnancy	Does not measure vaccine uptake, intent or hesitancy/acceptance during pregnancy
**More et al. 2017**	The Lancet Global Health	Community resource centres to improve the health of women and children in informal settlements in Mumbai: a cluster-randomised, controlled trial	Does not measure vaccine uptake, intent or hesitancy/acceptance during pregnancy
**O'Leary et al. 2016**	Pediatric Academic Societies Annual Meeting	A pragmatic cluster randomized trial to increase uptake of vaccines during pregnancy	Not a primary study [conference abstract]
**O'Leary et al. 2019**	Open Forum Infectious Diseases	Improving uptake of maternal immunizations in the obstetric care setting through an adaptation of the CDC's immunization quality improvement program (AFIX)	Not a primary study [conference abstract]
**Orgul et al. 2021**	Georg Thieme Verlag	The Rate of Influenza Vaccination after Face-to-Face Interview in Pregnancy	Not a randomized trial or quasi-experimental design
**Rasmawati et al. 2021**	Turkish Journal of Physiotherapy and Rehabilitation	The effect of husband's support media applications in the "mattampu" antenatal visits on husband's knowledge	Does not measure vaccine uptake, intent or hesitancy/acceptance during pregnancy
**Salmon et al. 2019**	Vaccine	MomsTalkShots: an individually tailored educational application for maternal and infant vaccines	Does not measure vaccine uptake, intent or hesitancy/acceptance during pregnancy
**Seo & Lim 2018**	Vaccine	Trends in influenza vaccination coverage rates in South Korea from 2005 to 2014: Effect of public health policies on vaccination behavior	Not a randomized trial or quasi-experimental design

**Sharma et al. 2023**	IEEE Journal of Biomedical & Health Informatics	SwasthGarbh: a smartphone App for improving the quality of antenatal care and ameliorating maternal-fetal health	Does not measure vaccine uptake, intent or hesitancy/acceptance during pregnancy
**Stringer et al. 2013**	BMC Health Services Research	Protocol-driven primary care and community linkages to improve population health in rural Zambia: the Better Health Outcomes through Mentoring and Assessment (BHOMA) project	Does not measure vaccine uptake, intent or hesitancy/acceptance during pregnancy
**Villadsen et al. 2013**	Tropical Medicine and International Health	Evaluation of a participatory antenatal care strengthening intervention on health promoting behaviours in Jimma, Ethiopia, 2009-2011	Does not measure vaccine uptake, intent or hesitancy/acceptance during pregnancy
**Walker et al. 2019**	Global Public Health	Impact of Muslim opinion leaders' training of healthcare providers on the uptake of MNCH services in Northern Nigeria	Does not measure vaccine uptake, intent or hesitancy/acceptance during pregnancy
**Younes et al. 2015**	Journal of Epidemiology & community Health	The effect of participatory women's groups on infant feeding and child health knowledge, behaviour and outcomes in rural Bangladesh: a controlled before-and-after study	Does not measure vaccine uptake, intent or hesitancy/acceptance during pregnancy
**Yudin et al. 2015**	American Journal of Obstetrics & Gynecology	Text message reminders do not increase the likelihood of influenza vaccination among pregnant women	Not a primary study [conference abstract]

Figure S1. Geographic location of studies identified that evaluate interventions to increase vaccine uptake during pregnancy.

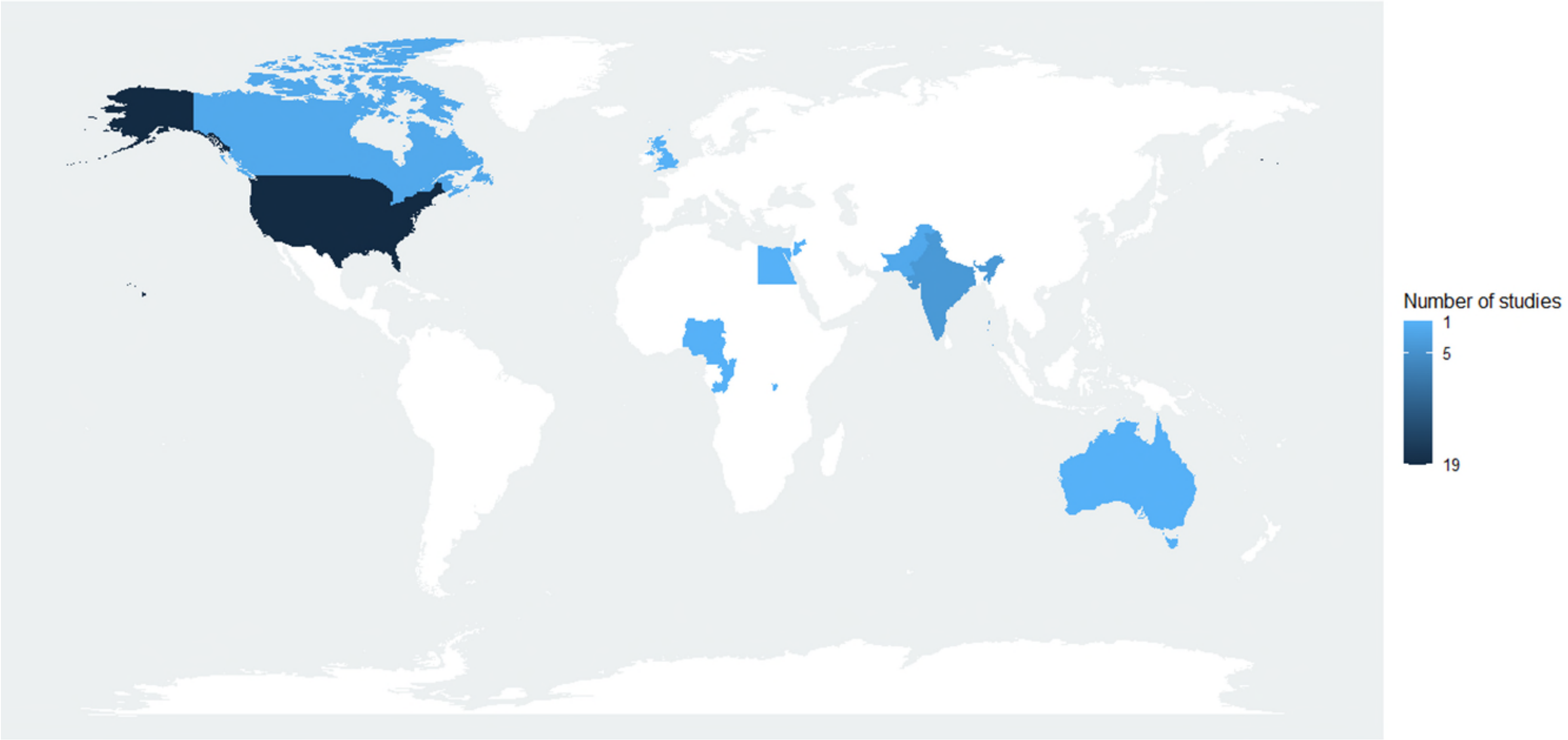

Table S2. Description of interventions to increase vaccine uptake during pregnancy.

**Author, Year**	**Intervention Type**	**Description of the Intervention(s)**
**Patient-level Interventions**		
Choudhury *et al.* 2021	Patient education	A Mobile Health Technology for Mothers (MFM) software application was developed by nongovernmental organizations. The app was designed for low-literacy users and could be run open source on a Java-enabled form or Android-based smartphone and was administered in Hindi. The application consisted of 4 modules: (1) registration, (2) antenatal care, (3) intra-natal care, and (4) postnatal care. In addition, the Interactive Voice Recording System enabled mHealth to provide maternal health information through texts, photographs, and voice prompts (in the user's native language) to pregnant women and mothers. The mHealth intervention was administered by accredited social health activists during 4 x prenatal visits (with first occurring in the first trimester) and 2 x postnatal visits (3rd and 6th months after childbirth). During each home visit, the accredited social health activist carried and used the mHealth application to implement on-to-one counseling with pregnant patients about maternal healthcare and hygiene, as appropriate for their stage of pregnancy. Each encounter lasted for approximately 45 minutes.
Dudley *et al.* 2022	Patient education	A web-based application (MomsTalkShots) designed to be accessible via multiple internet browsers on smartphones, tablets, and computers. The app begins with registration, then administers a survey, then immediately provides educational videos that are algorithmically responsive to its users' vaccine intentions, KABs, and demographics. The videos incorporate introductions and conclusions from obstetricians and pediatricians of different races/ethnicities with narrated animation to communicate messages in an interesting and engaging manner. The videos were designed based on approaches shown to be effective in training healthcare providers to improve their vaccine discussions with patients: for patients already intending to vaccinate, taking a presumptive approach; and for patients with concerns, establishing empathy, then carefully addressing the concerns within the context of the risk of disease, the benefits of vaccination, and the ability to protect through vaccinating. Videos covered both maternal and infant vaccination. Videos were also available in a gallery for rewatching later. A feature also allowed participants to refer the app to their close contacts.
Frew *et al.* 2016	Patient education and persuasive messaging	Two Elaboration Likelihood Model (ELM)-based education and persuasive messaging interventions were applied:

		**Intervention 1:** A “Pregnant Pause” video showed physicians providing detailed information on pertussis and influenza vaccines, the severity of pertussis and influenza, how the vaccines protect pregnant women and newborns, safety information, and the current ACIP recommendations. The video was given to participants in the waiting room, and if not completed before being called in for their appointment, they were permitted to take the iPad to the examination room to complete.<br><br>**Intervention 2:** A tutorial produced using the iBook platform that included text and audio/visual content covering the importance of vaccination during pregnancy, the dangers of influenza and pertussis to infants, safety of antenatal vaccination, recommended timing of antenatal vaccination, current ACIP recommendations for vaccination during pregnancy, and an introduction to childhood vaccination. Videos included obstetric physicians talking about antenatal vaccination. The tutorial provided information through an interactive question-and-answer format. Participants could choose the topic(s) that most interested them and complete each section of the tutorial separately. The iBook was given to participants in the waiting room, and if not completed before being called in for their appointment, they were permitted to take the iPad to the examination room to complete.
Goodman *et al.* 2015	Patient education	An educational online video that was developed by the Centers for Disease Control and Prevention (CDC), called *Protect Yourself, Protect Your Baby* (3 ½ minutes). The video addresses vaccination health beliefs concepts found to be predictive of vaccination and is intended to contain a clear and easy to understand format.
Jordan *et al.* 2015	Patient education and patient reminders	SMS education and usual or enhanced reminders were applied to two groups, based on responses to a baseline text message, enquiring whether they planned to be vaccinated this influenza season. Those who indicated they planned to be vaccinated were considered ‘planners’ and those who indicated they did not plan to receive a vaccine were considered ‘non planners.’<br><br>**Planner intervention:** Participants who replied by text that they were planning to be vaccinated were randomly assigned to receive either a ‘usual message’ group, where they received one encouragement message advising them to put a reminder on their calendar for influenza vaccination, or an enhanced reminder. The ‘enhanced messages’ group received one encouragement message as well as the opportunity to set up a general reminder (sent 2 weeks after receiving their response) or a specific reminder (sent 2 days before their chosen date).<br><br>**Non-planner intervention:** Participants responding at baseline that they were not planning to

		be vaccinated were randomly assigned to receive a usual message, which stressed the importance of influenza vaccination, or an enhanced message. The 'enhanced message' asked why they were not planning to be vaccinated with five options: *I think it may give me flu*, *Cost*, *Don't think it's safe*, *Don't need it*, and *Other*. An educational message tailored to the identified concern was then sent to each participant based on their response.
Kriss *et al.* 2017	Patient education and persuasive messaging	Two Elaboration Likelihood Model (ELM)-based education and persuasive messaging interventions were applied:<br><br>**Intervention 1:** A "Pregnant Pause" video showed physicians providing detailed information on pertussis and influenza vaccines, the severity of pertussis and influenza, how the vaccines protect pregnant women and newborns, safety information, and the current ACIP recommendations. The video was given to participants in the waiting room, and if not completed before being called in for their appointment, they were permitted to take the iPad to the examination room to complete.<br><br>**Intervention 2:** A tutorial produced using the iBook platform that included text and audio/visual content covering the importance of vaccination during pregnancy, the dangers of influenza and pertussis to infants, safety of antenatal vaccination, recommended timing of antenatal vaccination, current ACIP recommendations for vaccination during pregnancy, and an introduction to childhood vaccination. Videos included obstetric physicians talking about antenatal vaccination. The tutorial provided information through an interactive question-and-answer format. Participants could choose the topic(s) that most interested them and complete each section of the tutorial separately. The iBook was given to participants in the waiting room, and if not completed before being called in for their appointment, they were permitted to take the iPad to the examination room to complete.
Meharry *et al.* 2013	Patient education	**Group 1:** Received standard care and an educational pamphlet on influenza vaccine in pregnancy developed by the Hong Kong Centre for Health Protection (CHP), which is freely available in all antenatal clinics.<br><br>**Group 2:** Received standard care in addition to one-to-one, brief education that focused on four key recommendations identified from the research literature: (i) inform pregnant women of vaccination recommendations, (ii) encourage vaccine discussions with their HCPs, (iii) increase accessibility of vaccine (make referral to clinics where vaccine can be obtained), and (iv) provide credible information from the government official website and provide the website URL.

		Participants in the education group were informed about the: (i) global and national recommendations regarding influenza vaccine during pregnancy, (ii) potential complications associated with influenza infection during pregnancy and for young infants, (iii) safety of influenza vaccine for pregnant women and the fetus, and (iv) potential benefits of influenza vaccine for the pregnant women, the fetus and the infant, and (iv) where and how to get the influenza vaccine.
Momani *et al.* 2023	Patient education	Individual tele-education (interactive education phone sessions, phone calls consultancy, text message, and digital education booklet) provided to the intervention group for two weeks. Information provided in the educational program was adopted from the CDC. The researcher called participating women in the intervention group and discussed COVID-19 disease, the benefits of the vaccine, effectiveness, side effects of the available vaccines, sources of their information about the vaccine, and answered their questions about the disease and the vaccine. Participants in the intervention group also had the chance to contact the researcher asking for information/further explanation over the phone.
Moniz *et al.* 2013	Patient education	**Group 1:** Participants received 12 weekly text messages including text messages regarding general preventive health in pregnancy (i.e., importance of prenatal vitamins, nutritional foods, and seatbelt use) in addition to messages on the importance of influenza vaccination during pregnancy. (i.e., benefits and safety of influenza vaccination during pregnancy).<br><br>**Group 2:** Participants received only messages regarding general preventive health in pregnancy.
O'Leary *et al.* 2019	Patient education	**Group 1**: A website providing education on recommended vaccines during pregnancy and childhood immunizations. Information on the website included national vaccine recommendations during pregnancy, details on each recommended vaccine (including safety information and ingredients), a description of the diseases each vaccine prevents, and answers to common vaccine concerns during pregnancy<br><br>**Group 2**: A website providing education on recommended vaccines during pregnancy as well as childhood immunizations. Information included national vaccine recommendations during pregnancy, details on each recommended vaccine (including safety information and ingredients), a description of the diseases each vaccine prevents, and answers to common vaccine concerns during pregnancy, IN ADDITION TO<br>interactive website components including a blog, discussion forum, chat room, and an "Ask a Question" portal through which participants could ask experts questions about vaccination

Parsons *et al.* 2022	Patient education	A 4-minute animation video informed by Intervention Mapping which ensured that it was based on theory and evidence and grounded in the needs and preferences of the target population. The intervention addressed beliefs about the risk of influenza and the efficacy of the vaccination using animated messages and relatable characters.
Payakachat *et al.* 2016	Patient education	Participants were given a modified Tdap Vaccine Information Statement (VIS) created by the Plain Language Training Program using NIH guidelines for plain language and health literacy best practices.
Regan *et al.* 2017	Patient reminder	A SMS reminder was sent during influenza vaccine availability from the patients' primary care provider to remind unvaccinated patients to schedule an appointment for their influenza vaccine.
Sato & Fintan 2019		Participants were randomized to one of three groups in a conditional cash transfer program. Each participant received a voucher in the amount of:<br><br>(1) C5 naira (control group)<br>(2) C300 naira<br>(3) C800 naira<br><br>Participants were asked to bring the voucher to the clinic when they arrived for tetanus toxoid vaccination. The voucher indicated the respondents' names as well as the amount of cash incentive they could redeem. The voucher could be redeemed for cash at the clinic after vaccination.
Stockwell *et al.* 2014	Patient reminder	Text message reminders were used to remind pregnant patients about the need for influenza vaccination. A sequence of 5 weekly, automated text message influenza vaccine reminders were developed based on feedback from focus groups. The first message was introductory to let participants know they were due for an influenza vaccine. Three other messages provided educational information, including (1) that pregnant people and their newborns are at increased risk for influenza-related illness, (2) vaccine safety, and (3) that doctors recommend the influenza vaccine. Some messages suggested that participants discuss the vaccine at their next prenatal visit. The fifth message was interactive; participants could select to receive more information regarding influenza risk, common misperceptions regarding the influenza vaccines, side effects, and need for yearly influenza. A final message was sent to assess satisfaction with the text messaging service to which participants were asked to reply whether they "really liked" the messages, thought they were "ok," or "did not like" them. The messages were written at a

		fourth-grade level per the Flesch-Kincaid readability statistic.
Wong *et al.* 2016	Patient education	Brief one-on-one education lasting 10 min that focused on four key recommendations identified from the literature: (i) informing the participants about vaccination recommendations; (ii) encouraging them to discuss vaccination with their antenatal care provider or general practitioner (GP); (iii) increasing accessibility of the vaccine by referral to clinics where vaccination could be obtained; and (iv) providing influenza-related information from the official government website and the website uniform resource locator.
Yudin *et al.* 2017	Patient reminder	Targeted electronic reminders (text messages) twice weekly for four weeks for a total of eight messages + an educational pamphlet. Messages focused on influenza and the influenza vaccine and were developed using principles from the Health Belief Model (i.e., emphasizing susceptibility of pregnant women to influenza, effectiveness of the vaccine for decreasing disease, safety of vaccine, and recommendations).
**Provider-level interventions**		
Klatt & Hopp 2012	Provider reminder	At the beginning of the influenza season, providers at the participating clinic were reminded of the availability of influenza vaccines and the importance of vaccinating every pregnant patient. A best-practice alert was incorporated into the electronic medical record system. The electronic reminder alerted the health care provider during each prenatal visit if the patient's medical record did not yet contain documentation of an influenza vaccination. Once the patient received a vaccine, the alert no longer appeared at subsequent visits. The alert could be satisfied by 1) ordering the vaccine, 2) documenting that a vaccine had been given elsewhere, or 3) by selecting "declined" and providing a reason.
Woolley *et al.* 2018	Provider education	A socially accountable health professional education program (SAHPE) for medical schools, including community-engaged learning components. All institutions employing SAHPE programs selected students who more accurately reflect the geographical, ethnic and socio-economic diversity of the school's reference population. All SAHPE institutions use extended community-engaged service learning in local communities and a locally relevant curriculum to produce graduates trained in regional health priorities. Students are based in rural communities for over one year of their training, during which time they complete significant community service and public health activities. Curriculum activities train students in public health, community development strategies, and the diagnosis and treatment of key local health issues. This approach aims to develop a commitment to community service and a willingness to serve disadvantaged population subgroups or communities regardless of socio-economic or ethnic

<table>
<tr><td></td><td></td><td>divisions.</td></tr>
<tr><td colspan="3">Systems-level interventions</td></tr>
<tr><td>Balakrishnan et al. 2016</td><td>mHealth service</td><td>A mHealth platform to support continuum of care services for maternal and child health. Components of the platform included:

(1) Home visit planner:
(a) Registration and name-based tracking of beneficiaries – pregnant women and children below 6 years of age;
(b) Automatic scheduling of home visits for the frontline workers;
(c) Automatic generation of list of children due for immunization;
(d) Interactive checklist and counseling protocols to promote and gather maternal and child health in the continuum;
(2) Animated videos as job aids for providing interpersonal communication;
(3) Tools to compute expected date of delivery, body mass index, child growth standards;
(4) Convergence and case sharing between health (MoHFW) and nutrition (MoWCD) departments at service delivery level;
(5) Data driven supportive supervision for periodical review and monitoring;
(6) Synchronization of frontline worker activities with supervisor to enable indicators-based monitoring;
(7) Activities based approach and effortless data recording of each beneficiary in the continuum.

The platform was used by frontline community health workers. The platform includes scheduling and checklist functions with additional multimedia components to help interpersonal communication by the frontline worker. A central database keeps patient data synchronized between mobile phone, the supervisor’s mobile phone, and the central database, allowing improved monitoring and supported supervision. The platform additionally facilitated integration of work by accredited social health activists and Anganwadi workers - the two primary providers of maternal and child health services.

Pregnant patients and newborns were entered as a case into the system and followed with care provided until the child was 6 years old.</td></tr>
<tr><td>Bonfrer et al. 2014</td><td>Performance-based</td><td>Performance-based financing by the federal government, supplemented by the World Bank and</td></tr>
</table>

	financing	other donors. Facilities receive payments based on quantity and quality-based outcomes.<br><br>Quantity-based payments were measured through twenty-three output indicators. For this study, data were collected for six of these output indicators. Health care facilities reported monthly to the Ministry of Health about quantities of health services delivered for each indicator. Reported quantities were verified and validated by a provincial committee through unannounced observation visits to facilities.<br><br>Facilities could receive a quality bonus of up to 25 percent. Quality was assessed quarterly by local regulatory authorities on a randomly chosen day using a checklist containing 220 items grouped into the following topics: general infrastructure and communication, business plan, income and costs, hygiene and sterilization, outpatient consultations, family planning, laboratory services, inpatient care, management of essential drugs, availability of essential drugs, maternal care, surgery, tuberculosis screening, vaccination, and antenatal care.<br><br>The total payment to a facility is calculated as a weighted sum of the number of provided services in the previous three months times their unit payment multiplied by the quality bonus, which ranged between 1 and 1.25 depending on the score obtained from evaluation of facilities based on results of the checklist assessment.
Chakrabarti *et al.* 2021	Conditional cash transfer	Conditional cash transfer program targeting pregnant and lactating women aged ≥19y, providing a financial incentive of INR 5000 (~US $70; equivalent to 5% of the average per capita income in Odisha) dependent on utilization of maternal healthcare services.<br><br>The first installment of INR 3000 in the third trimester is contingent on (1) pregnant person being registered within 3-4 months of pregnancy; (2) beneficiary opening a bank account; (3) ≥2 ANC visits, receipt of IFA tablets, receipt of ≥2 tetanus vaccination, and receipt of ≥1 counseling session from a HCW.<br><br>The second installment of INR 2000 is contingent on (1) childbirth being registered, (2) completing immunization according to national guidelines, (3) attendance at 6 growth monitoring and feeding counseling sessions, and (4) initiation and continuation of age-appropriate complementary feeding after 6 months.
de Walque *et al.* 2021	Performance-based financing	**Performance-based financing:** A performance-based financing program was implemented by Performance Purchasing Agencies (PPAs), who were autonomous entities contacted by the

		government to generate performance contracts with health facilities, verify health facility data, and manage performance-based funding. Contracts with facilities were signed for three months. Health facilities were responsible for completing registers with information on the number of patients seen for each health service and monthly activity reports and declaration forms. The performance-based finance program incentivized curative care (e.g., outpatient consultations, hospitalizations and minor surgeries), preventive care (e.g., vaccination, infectious disease screenings) and reproductive healthcare (e.g., deliveries, family planning, antenatal care (ANC) services).<br><br>Following verification, performance payments were provided monthly and included a fixed amount for each health service provided that was scaled by a quality score and equity bonus. The equity bonus advantaged poorer and more remote health districts. Quality assessments were conducted quarterly and based on a standardized checklist measuring structural quality of care (i.e., facility management, hygiene, sanitation) as well as certain attributes of service delivery (i.e., correct case management). Facilities were provided with the same supervision and monitoring and managerial autonomy over the budgetary supplement received. Facilities had autonomy to hire staff with their performance-based revenue and to fire staff if necessary.<br><br>**Direct financing:** Facilities received a fixed per capita budgetary supplement unrelated to performance that matched the per capita budgetary allocation for the facilities. Facilities were provided with the same supervision and monitoring and managerial autonomy over the budgetary supplement received. Facilities had autonomy to hire staff with their budgetary supplement received and to fire staff if necessary.<br><br>**Enhanced supervision and monitoring:** Facilities received no additional resources and received the same supervision and monitoring as in the performance-based financing and direct financing facilities.
Li *et al.* 2022	Models of vaccine delivery	Four models of pertussis vaccine delivery for pregnant patients were evaluated:<br><br>**Local community service centers model:** A nurse recruited participants into a family medicine group clinic and referred them to their local community service center for vaccination administration. Community service centers offer community health care services and serve a specific geographical area. This was the existing standard for pertussis vaccination in pregnant patients.<br><br>**Family medicine group model:** On the day the vaccine was recommended, participants were

		offered pertussis vaccination at the family medicine group where their pregnancy was followed by a team of family physicians and nurses.<br><br>**Obstetrics clinic model:** Participants were recommended pertussis vaccine at an obstetrics appointment at a high-speed obstetrics follow-up clinic and a nurse offered the vaccine at a separately scheduled nursing appointment in the same clinic.<br><br>**Oral glucose challenge test model:** At the time of a routinely scheduled gestational diabetes screening between weeks 24 and 28 of gestation conducted at a mother and child teaching hospital, patients were offered pertussis vaccination during the wait hour of the same appointment or after blood procurement. Those with pre-existing diabetes were referred to local community service centers for vaccination.
Mouzoon *et al.* 2010	AFIX intervention	The multi-component intervention included three components:<br><br>(1) **Feedback**: Baseline immunization rates for each obstetrician's pregnant population were assessed. Information on individual and departmental rates was provided to each obstetrician. This assessment was followed by direct encouragement to immunize according to ACIP guidelines in any trimester of pregnancy. The chief of the obstetrics and gynecology department (FAS) served as an immunization champion, modeling behaviors such as encouraging nurse assessment and promotion of immunization and immunizing patients at every opportunity. Educational updates on influenza vaccination in pregnancy and regular rate assessments were provided to obstetricians, nurses, and staff during quarterly meetings.<br><br>(2) **Standing orders**: for influenza vaccine administration were revised to specifically encourage immunization in pregnancy by all immunization providers (primary care physicians and immunization nurses). Training on their use was provided to obstetricians, obstetric nurses, and immunization nurses.<br><br>(3) **Training**: obstetric nurses were offered training on vaccinations and were encouraged to take the initiative in identifying and completing immunization for eligible patients. Strong departmental leadership and physician interest in best practices led to the routine practice of nurses' taking responsibility for immunization of pregnant patients under standing orders for most.
Mwase *et al.* 2022	Performance-based financing	Four different models of performance-based financing (PBF) were evaluated:

		**Standard PBF Model 1:** Performance contracts based on case-based payments method adjusted for quality were signed between the Ministry of Health and health facilities. Verification agencies were employed to verify service provision data submitted by individual facilities. PBF unit prices were calculated based on the relative cost and frequency of the services provided. Additional incentives were calculated based on quantity outcomes and service quality if facilities achieved a quality score of 50% (and later changed to 60%), every quarter. Incentives were expected to pay for expenditures incurred, to increase savings and to pay bonuses to individual staff members.<br><br>**PBF Model 2 (PBF 1 + systematic targeting of ultra-poor):** The model used the same health service purchasing model as PBF1, but had specific equity measures meant to ease access to and utilization of maternal healthcare services among the ultra-poor living in the catchment areas of the participating health facilities with the following components: (a) a systematic targeting of the ultra-poor to identify a maximum of poorest 20% of the population; (b) providing the identified ultra-poor with proof of status so they could access health services at no cost at the point of use; and (c) higher purchase unit prices than in PBF1 for health services delivered to the targeted ultra-poor (i.e., as compensation for the lost revenues due to free health services provided to the ultra-poor at the point of use). The adjusted higher unit prices were only for services where user fees existed, such as tetanus toxoid vaccine, delivery and family planning services among others, while for services already provided free of charge at point of use, such as HIV and tuberculosis testing and treatment among others, the same unit prices as in standard PBF were used. The additional payments were removed in June 2016 after the introduction of the national free healthcare policy.<br><br>**PBF Model 3 (PBF Model 2 + higher incentive purchase price):** The model used the same purchasing arrangement as PBF1 and PBF2 and also involved the same targeting mechanisms and equity measures for the ultra-poor as in PBF2. The main difference was in the unit prices, whereby services provided to the ultra-poor were reimbursed at a higher unit price than in PBF2—at around 150% of the PBF2 unit prices. The higher unit prices were meant to compensate for the lost revenue from user fees, and also to offer health workers an additional incentive to motivate them to attract or reach out to the ultra-poor. This applied only to services where user fees were still charged at the point of use. These additional payments were removed in June 2016 after the introduction of the national free healthcare policy.

		**PBF Model 4 (PBF 1 + community-based health insurance and targeted and subsidization of health services for ultra-poor):** The model implemented PBF1 alongside community-based health insurance, whereby an annual insurance premium of 3900 F CFA (equivalent to US$7) per individual was offered for the whole population using the same targeting mechanism as in PBF2 and PBF3.
Shah *et al.* 1993	Home-based maternal health record	Use of a home-based maternal record (HBMR) to record risk factors and early signs or symptoms of complications (i.e., pallor, oedema, bleeding, weight, abdomen size, presentation, fetal movements). Data are entered by community health workers, clinic staff, traditional birth attendants, or the mother herself. The HBMR can be linked with center-based records and can provide constantly available information and improve continuum of care throughout pregnancy, labor, delivery, postpartum, and intrapartum.<br><br>The HBMR empowers women to recognize and understand health problems and participate in the improvement of their own health and that of their newborn infant.
Wootton *et al.* 2018	Opt-out vaccine delivery	Patients received information regarding influenza infection and vaccination and the standard Vaccine Information Statement. Following this, patients were asked to complete a consent document to opt out of influenza immunization.
Zeng *et al.* 2018	Performance-based financing	A results-based financing scheme (Cordaid) was implemented. Cordaid monitored monthly reports of quantities of services and verified these against facility registers. Households were sampled to assess the quality of services received. The amount of payment was dependent on the service. Incentive payments were made in addition to the routine budget allocated by the Ministry of Health and Population. Cordaid also paid financial incentives to technical teams in each district that were responsible for supervising and helping health centers identify service provision issues and suggesting solutions.
**Patient, Provider, & Systems-level Interventions**		
Brewer *et al*. 2019	**Systems-level:**<br>Standing orders; provision of vaccines on-site; enhanced vaccine documentation; immunization champion; non-provider staff education; AFIX<br><br>**Provider-level:** Provider	A multimodal intervention that included components addressing patient, provider and systems-level:<br><br>**Systems-level**<br>(1) **Stocking of influenza and pertussis vaccines** (*if participating practices did not already*)<br><br>(2) **Standing orders**: Practice-wide rules allowing medical assistants or nurses to deliver vaccines

	education; provider feedback<br><br>**Patient-level:** Patient education	to eligible patients without individual physician orders. Standing orders for vaccines already in place were revised or expanded depending on the vaccine. The electronic clinical decision support tool is queried for each patient as they are triaged for a clinic visit for vaccine standing orders. Prior to the intervention, this tool would only provide a standing order for a vaccine if the vaccine was covered by insurance. This was revised to provide an order for all indicated vaccines regardless of insurance. The standing orders were also expanded to include influenza in the outpatient setting, as previous to the intervention there was only a standing order for influenza vaccine on the inpatient wards. All practice staff were trained in standing order implementation.<br><br>(3) **Immunization champion**: A registered nurse who performed periodic chart reviews and gave immediate feedback to providers when missed opportunities were identified. Immunization champions met biweekly with study staff to receive implementation support and technical assistance from study staff and to report on fidelity of implementation throughout the intervention phase of the study.<br><br>(4) **Incentives:** At each clinic, a medical assistant who had the highest rates of vaccination prior to the intervention was given recognition at her clinic by receiving institutional awards.<br><br>(5) **Standardized immunization documentation:** Practice-specific EHR documentation protocols were developed and implemented with the aim to improve vaccine documentation in patient records.<br><br>**Provider-level**<br>(1) **Provider education:** Practice staff were provided with standardized education regarding ACIP recommendations<br>(2) **Provider feedback:** Providers at participating practices received regular feedback on performance from the immunization champion.<br><br>**Patient-level**<br>(1) **Patient education:** Patients routinely received handout educational materials on influenza and pertussis vaccines at each prenatal visit and at ultrasound visits.
Chamberlain *et al.* 2015; Chamberlain *et al.* 2016	**Systems-level:** Immunization champion	A multi-component intervention package including patient, provider, and practice level components:

	**Provider-level:** Provider education<br><br>**Patient-level:** Patient education; map to local vaccine sites	**Practice-level components**<br>(1) **Vaccine champion:** A staff member identified by the practice to be the primary resource for vaccine-related information for all staff. The individual could hold any position but needed a positive attitude about vaccination and be willing to promote the intervention throughout its duration.<br><br>(2) **Lapel buttons:** All staff were encouraged to wear buttons promoting antenatal vaccination on the lapels of their jackets or scrubs.<br><br>(3) **Posters:** Posters promoting antenatal influenza and pertussis vaccination. Posters were hung in prominent places in the office, including waiting rooms, exam rooms, restrooms and hallways.<br><br>(4) **Brochures:** Educational brochures emphasizing the importance of antenatal vaccination, the composition of influenza and pertussis vaccines, the safety of vaccines, vaccines, and the recommended timing of vaccination.<br><br>**Provider-level components**<br>(1) **Provider-to-patient talking points:** Talking points for how to promote antenatal influenza and Tdap vaccination were provided to vaccine champions. Three primary talking points that emphasized protection of the fetus and newborn were produced for each vaccine, followed by additional safety-related talking points.<br><br>(2) **Peer-to-peer vaccine promotion education:** During a one-hour lunch session, the Georgia Educating Physicians in their Communities (EPIC) program implemented a peer-to-peer vaccine promotion education session. The session was led by a nurse or physician and covered the importance of antenatal vaccination, tips for starting an in-house vaccination program, and financial aspects of managing vaccines in an obstetric setting.<br><br>**Patient-level components**<br>(1) **iPad-based interactive tutorial:** A tutorial produced using the iBook platform that included text and audio/visual content covering the importance of vaccination during pregnancy, the dangers of influenza and pertussis to infants, safety of antenatal vaccination, recommended timing of antenatal vaccination, and an introduction to childhood vaccination. Videos included obstetric physicians talking about antenatal vaccination in addition to testimonials from mothers of infants who contracted influenza and pertussis.

		(2) **Maps to local pharmacies/health departments that provide vaccines** (*for practices that did not offer one or both vaccines only*): A list and map of health departments and retail outlets within 5-10 miles of a practice, including facility address and contact information, distance from practice, maximum price of vaccine, and whether the facility would file insurance claims.
Dehlinger *et al.* 2021	**Patient-level**<br>Patient education<br><br>**Provider-level**<br>Provider education, Provider reminders	**Patient-level**<br>(1) **Patient education:** Evidence-based education provided via a one-page opt-in or opt-out vaccination consent form. When signed, the form documented the patient's acceptance or refusal of the vaccine. The consent form was developed with information from the CDC's website for influenza vaccines for pregnant people. It was designed to address myths and inform patients of the infant and maternal benefits of influenza vaccination during pregnancy. Posters with similar information encouraging vaccination were developed and provided by the CDC and were placed in patient restrooms.<br><br>**Provider-level**<br>(1) **Provider education:** At the beginning of influenza vaccine availability, clinicians and medical assistants were educated during monthly staff meetings regarding the importance of an earnest recommendation from a health care provider, positive message framing, and known patient barriers to vaccination. They were also provided with a laminated copy of appropriate ICD-10-CM codes to document influenza vaccine administration, declining of influenza vaccine, receipt of influenza vaccine elsewhere, and whether a patient's vaccination status is up-to-date. Staff were encouraged to use these codes in patient medical records. Clinicians and medical assistants not present at staff meetings were counseled individually over a two week period.<br><br>(2) **Periodic reminders:** Three emails with information from the CDC's website were sent to staff periodically during the season. Emails included information on the importance of making a strong recommendation for influenza vaccination, current trends in influenza infection data, and a weekly influenza surveillance report. A best practice advisory alert was built into the electronic health record system. The electronic reminder alerted the health care provider during each prenatal visit if the patient's medical record did not yet contain documentation of an influenza vaccination. Once the patient received a vaccine, the alert no longer appeared at subsequent visits. The alert could be satisfied by 1) ordering the vaccine, 2) documenting that a vaccine had been given elsewhere, or 3) by selecting "declined" and providing a reason. Dismissal of the alert could occur if documentation of vaccination status was not feasible.
Mozzani *et al.* 2016	**Systems-level:**	A multimodal intervention that included components at the patient, provider and systems

	Standing orders; vaccines on-site; immunization champion; non-provider staff education<br><br>**Provider-level:** Provider education; provider feedback<br><br>**Patient-level:** Patient education	levels, including:<br><br>**Systems-level**<br>(1) **Stocking of influenza and pertussis vaccines** (*if participating practices did not already*)<br><br>(2) **Standing orders**: Practice-wide rules allowing medical assistants or nurses to deliver vaccines to eligible patients without individual physician orders. Standing orders for vaccines already in place were revised or expanded depending on the vaccine. The electronic clinical decision support tool is queried for each patient as they are triaged for a clinic visit for vaccine standing orders. Prior to the intervention, this tool would only provide a standing order for a vaccine if the vaccine was covered by insurance. This was revised to provide an order for all indicated vaccines regardless of insurance. The standing orders were also expanded to include influenza in the outpatient setting, as previous to the intervention there was only a standing order for influenza vaccine on the inpatient wards. All practice staff were trained in standing order implementation.<br><br>(3) **Immunization champion**: A registered nurse who performed periodic chart reviews and gave immediate feedback to providers when missed opportunities were identified. Immunization champions met biweekly with study staff to receive implementation support and technical assistance from study staff and to report on fidelity of implementation throughout the intervention phase of the study.<br><br>(4) **Incentives:** At each clinic, a medical assistant who had the highest rates of vaccination prior to the intervention was given recognition at her clinic by receiving institutional awards.<br><br>(5) **Standardized immunization documentation:** Practice-specific EHR documentation protocols were developed and implemented with the aim to improve vaccine documentation in patient records.<br><br>**Provider-level**<br>(1) **Provider education:** Practice staff were provided with standardized education regarding ACIP recommendations<br>(2) **Provider feedback:** Providers at participating practices received regular feedback on performance from the immunization champion.<br><br>**Patient-level**<br>(1) **Patient education:** Patients routinely received handout educational materials on influenza

		and pertussis vaccines at each prenatal visit and at ultrasound visits.
Ohly *et al.* 2018	**Systems-level:** service delivery; staff capacity development; community engagement<br><br>**Patient-level:** conditional cash transfer	The intervention was designed with community input via household surveys, rapid needs assessments, and meetings with community leaders. The final intervention was multifaceted, with four main components:<br><br>**Systems-level**<br>(1) Service development including reproductive health, immunizations, gynecological, safe delivery and nutrition services;<br><br>(2) Staff capacity development including professional staff and volunteers;<br><br>(3) Community engagement including the formation of village health committees (locally known as Jirga) to promote awareness and assist with health campaigns and referrals to the health center; and<br><br>**Patient-level**<br>(4) Introduction of a micro-credit scheme to provide financial support to pregnant women (equivalent to 12 GBP) to cover the cost of ultrasound, transport expenses, medicine, and delivery charges.
Omer *et al.* 2022	**Systems-level:**<br>Standing orders; immunization champion; enhanced vaccine documentation; AFIX<br><br>**Provider-level:** Provider education<br><br>**Patient-level:** Patient education; map to local vaccine	A multi-component intervention package including patient, provider, and practice level components:<br><br>**Practice-level**<br>**(1) Immunization champion:** A staff member was selected by each participating practice to serve as a champion to increase vaccination. The champion provided encouragement, feedback, and promoted competition. Champions assisted in the implementation of all other practice-level interventions, including the provision of technical assistance from research staff through regular meetings, recruitment of participants, and measurement of study outcomes. Immunization champions were compensated $250 per year for their involvement.<br><br>(2) **AFIX visits:** An evidence-based quality improvement program originally designed to increase childhood immunization rates in public and private pediatric practice. A model adapted for obstetric practices was implemented, where baseline vaccination rates were evaluated, reviewed and shared with practice staff to set goals for improvement vaccination rates and to

		select appropriate interventions for each practice (*Feedback*). Interventions that could be implemented included standing orders, updating electronic health record protocols, educational flyers and other communication materials for patients and providers. Food was provided at all related meetings and providers could earn Maintenance of Certification credits through the American Board of Obstetrics and Gynecologists for participating in the AFIX process and completion of a provider-level education module (*Incentive*). After six months, change in immunization rates was evaluated and shared with practice staff in a follow-up session (*eXchange*).<br><br>**Provider-level**<br>(1) **Provider education:** A continuing medical education module (VaxChat) was developed by the study team based on five behavioral constructs, including disease salience, myth correction, nudge theory, presumptive communication, and motivational interviewing. The VaxChat module was completed by all providers at each intervention practice. In addition to the VaxChat module, written materials were given to all providers, including a copy of *A Clinician's Guide to Vaccine Safety*. The guide reviewed the US vaccine safety systems, described an approach to discuss vaccines with patients, and summarized each vaccine-preventable disease, and provided detailed vaccine recommendations for routinely recommended vaccines during pregnancy and infancy. Written materials also included a succinctly written review of 46 vaccine safety controversies with clear conclusions if the vaccine did or did not cause the adverse event. Talking points were included for each vaccine and vaccine safety issues.<br><br>**Patient-level**<br>(1) **Patient education:** Provision of a theory-driven, individually tailored app called MomsTalkShots, which administered video messages to address gaps, concerns, and baseline vaccine intent based on responses to a baseline questionnaire. Participants could receive up to six tailored videos depending on their concerns.
Spina *et al.* 2020	**Systems-level:** Immunization champion; standing orders; enhanced vaccine documentation; AFIX<br><br>**Provider-level:** Provider education	A version of the Assessment-Feedback-Incentive-eXchange (AFIX) model modified for quality improvement in obstetric care. AFIX-OB including four components:<br><br>(1) **Assessment:** Baseline vaccination rates for influenza and pertussis vaccination were obtained from manual chart reviews at each practice. An immunization delivery scale was used to collect data about vaccine delivery procedures at each practice, which aims to reveal three common shortfalls: (1) lack of utilization of Vaccine Information Sheets and/or education materials addressing vaccine concerns; (2) limited routine and systematic electronic health record or paper-based vaccine tracking protocols, and (3) lack of formal standing orders to

		reduce missed opportunities to vaccinate. These gaps informed which of the quality improvement strategies would be most applicable at the participating practice.<br><br>(2) **Feedback:** Baseline vaccination rates were presented to providers and staff at each practice, using a “maternal immunization report card” which compared baseline practice-specific maternal immunization rates to other obstetric sites, state and national rates and Health People 2020 goals. Key decision-makers were encouraged to attend a one-hour Feedback session at each clinic. Results of both the ”maternal immunization report card” and immunization delivery scale were presented at these meetings. Shortly following, practices set a goal to either increase their rates by at least 5 percentage points (if baseline rates were <80% coverage) or to increase their rates by at least 1 percentage point (if baseline rates were >=80% coverage). Each practice committed to at least one quality improvement measure to implement over the next six months.<br><br>(3) **Incentives**: Monetary incentives were provided to immunization champions in the amount of $500 over a two-year commitment in exchange for assisting in the implementation of AFIX-OB. Immunization champions additionally received biweekly technical assistance and support. Food incentives were provided for staff at the initial feedback meeting and follow-up eXchange meeting to incentivize attendance. Finally, providers who participated in the initial meeting, completed and education continuing medical education module, and adhered to the six-month intervention commitment were eligible to receive American Board of Obstetrics and Gynecology Maintenance of Certification credits.<br><br>(4) **eXchange:** Practice vaccination rates were reassessed after six months, which included updating the “maternal immunization report card” and presenting results at a follow-up meeting. Follow-up data were presented in 15-20 minute in-person meetings with practice providers, staff, and immunization champions. A “sustainability package” was developed that provided suggestions to encourage successful progression toward unmet goals or maintenance of successfully implemented strategies.

Table S3. Ongoing registered clinical trials aiming to increase the uptake of vaccines recommended during pregnancy.

**Principal Investigator / Affiliation**	**Registration ID**	**Country**	**Vaccine Target**	**Title**	**Sponsor**	**Patients**	**Intervention**	**Comparator**	**Primary outcome measure(s)**
Kelly / Washington University School of Medicine	NCT06640868	USA	RSV vaccine	Pilot Sequential Multiple Assignment Randomized Trial for RSV Vaccine Uptake in Pregnancy	Washington University School of Medicine	Adult patients 18 years and older with a confirmed intrauterine pregnancy at 28-30 weeks with no contraindications for RSV vaccine	1) MyChart nurse message at 34 weeks<br>2) SMFM video education at 34 weeks<br>3) Visual aide encouraging RSV vaccination at 34 weeks<br>4) MyChart nurse message at 28-30 weeks<br>5) SMFM video education at 28-30 weeks<br>6) Visual aid encouraging RSV vaccination at 28-30 weeks	Usual care	RSV vaccination during pregnancy (32 weeks to birth)
Rand / University of Rochester	NCT04444518	USA	Influenza and pertussis vaccine	The VAX-MOM Study: Increasing Influenza and Tdap Vaccination of Pregnant	University of Rochester	Pregnant females eligible for influenza or pertussis vaccine (no age	A multi-component behavioral intervention (VAX-MOM) comprised of training in	Usual care	Influenza vaccination during pregnancy<br><br>Pertussis vaccination

				Women		restrictions)	communication, provider prompts, standing orders, and feedback on vaccination rates		during pregnancy
Patrizia / Fondazione Policlinico Universitario Agostino Gemelli IRCCS	NCT05785078	Italy	Influenza vaccine	Evaluation of a Flu Vaccination Program Directed at Pregnant Women Assisted at a Research Hospital in Italy	Fondazione Policlinico Universitario Agostino Gemelli IRCCS	Pregnant people and their partners attending the childbirth preparatory course held at Fondazione Policlinico Universitario "A. Gemelli" IRRCS	Educational session about maternal and childhood vaccinations	Pre-intervention	Knowledge and attitudes regarding vaccination
O'Leary / University of Colorado	NCT04302675	USA	Influenza and pertussis vaccine	Adapting Motivational Interviewing for Maternal Immunizations (MI4MI)	University of Colorado, Denver	Pregnant patients 15 years and older who are attending a participating OB-GYN practice who are eligible for maternal immunizations	Motivational interviewing, allowing providers to gain context, knowledge, confidence, and skills in talking to pregnant mothers about vaccination during pregnancy, and patients to have increased knowledge about	Pre-intervention	Influenza vaccination during pregnancy<br><br>Pertussis vaccination during pregnancy

							immunization issues and decreased anxiety about getting vaccinated		
Rand / University of Rochester	NCT05570630	USA	COVID-19 vaccine	VAX-MOM COVID-19: Increasing Maternal COVID-19 Vaccination	University of Rochester	Pregnant females eligible for COVID-19 vaccine (no age restrictions)	A multi-component behavioral intervention (VAX-MOM) comprised of training in communication, provider prompts, standing orders, and feedback on vaccination rates	Usual care	COVID-19 vaccination during pregnancy
Stefania / Fondazione Policlinico Universitario Agostino Gemelli IRCCS	NCT05729191	Italy	COVID-19 vaccine	Evaluation of the Acceptance of the Anti-COVID-19 Vaccine Offer and of the Knowledge and Attitudes Towards the Vaccination Among Pregnant Women Through a Validation Study at the Fondazione	Fondazione Policlinico Universitario Agostino Gemelli IRCCS	Pregnant people 18-50 years old attending the childbirth preparatory course held at Fondazione Policlinico Universitario “A. Gemelli” IRRCS	Anti-COVID-19 vaccine awareness event followed by a vaccine administration day	Pre-intervention	Acceptance of COVID-19 vaccination during pregnancy

				Policlinico Universitario A IRCCS Twins (FPG).					
Anraad / Maastricht University	NL-OMON25018	Netherlands	Pertussis vaccine	Effectiveness of a Centering Pregnancy and Online Tailoring intervention to promote informed decision making about maternal pertussis vaccination among pregnant women	ZonMW, The Netherlands Organisation for Health Resear	Pregnant people living in the Netherlands at 16 weeks gestational age or less, with good command of the Dutch language and access to the internet (no age restrictions)	(1) A Centering Pregnancy (CP) intervention during which maternal pertussis vaccination is discussed.<br><br>(2) An online tailored (OT) decision aid assisting in maternal pertussis vaccine decision-making	Not disclosed	Pertussis vaccine uptake during pregnancy

* Uptake is included as a secondary outcome only; process outcomes of implementing the intervention are included as the primary outcome measure.

Figure S2. Effect of patient education interventions to increase the uptake of recommended vaccines during pregnancy, by recommended vaccine.

Vaccine Target / Study	Country	Intervention Target	Study Design	Intervention Group Vaccinated	Intervention Group Total	Control Group Vaccinated	Control Group Total	RR	[95% CI]	Weight
**Influenza vaccine**										
Brewer et al 2019	USA	Systems−level	Historical control	49	150	27	125	1.51	[1.01; 2.27]	3.2%
Chamberlain et al. 2015	USA	Systems, Provider, & Patient	Cluster RCT	16	149	11	151	1.47	[0.71; 3.07]	1.6%
Dehlinger et al. 2021	USA	Systems, Provider, & Patient	Historical control	940	1487	870	1480	1.07	[1.01; 1.14]	6.1%
Frew et al. 2016	USA	Patient	RCT	4	31	4	34	1.10	[0.30; 4.02]	0.6%
Frew et al. 2016	USA	Patient	RCT	2	30	4	34	0.57	[0.11; 2.88]	0.4%
Goodman et al. 2015	USA	Patient	RCT	15	53	13	52	1.13	[0.60; 2.14]	1.9%
Jordan et al. 2015	USA	Patient	RCT	219	1025	267	1228	0.98	[0.84; 1.15]	5.4%
Meharry et al. 2014	USA	Patient	RCT	31	36	23	49	1.83	[1.32; 2.54]	3.9%
Meharry et al. 2014	USA	Patient	RCT	35	48	23	49	1.55	[1.10; 2.19]	3.7%
Moniz et al. 2013	USA	Patient	RCT	34	104	31	100	1.05	[0.71; 1.58]	3.3%
O'Leary et al. 2019	USA	Patient	RCT	80	140	16	44	1.57	[1.04; 2.38]	3.2%
O'Leary et al. 2019	USA	Patient	RCT	59	105	16	44	1.54	[1.01; 2.37]	3.1%
O'Leary et al. 2019	USA	Patient	RCT	80	140	59	105	1.02	[0.81; 1.27]	4.8%
Omer et al. 2022	USA	Systems, Provider, & Patient	RCT	347	523	320	525	1.09	[0.99; 1.19]	5.9%
Omer et al. 2022	USA	Patient−level	RCT	323	520	320	525	1.02	[0.93; 1.12]	5.9%
Yudin et al. 2017	Canada	Patient	RCT	40	129	41	152	1.15	[0.80; 1.66]	3.6%
Wong et al. 2016	Hong Kong	Patient	RCT	34	161	16	160	2.11	[1.22; 3.67]	2.3%
**Random effects model**								**1.21**	**[1.08; 1.35]**	**58.9%**
Heterogeneity: $I^2 = 51.4\%$, $\tau^2 = 0.0263$, $p = 0.007$										
**Pertussis vaccine**										
Brewer et al 2019	USA	Systems−level	Historical control	78	150	12	125	5.42	[3.10; 9.48]	2.3%
Chamberlain et al. 2015	USA	Systems, Provider, & Patient	Cluster RCT	19	140	13	151	1.58	[0.81; 3.07]	1.8%
Kriss et al 2017	USA	Patient	RCT	2	31	2	34	1.10	[0.16; 7.32]	0.3%
Kriss et al 2017	USA	Patient	RCT	2	30	2	34	1.13	[0.17; 7.56]	0.3%
Mazzoni et al. 2016	USA	Systems, Provider, & Patient−level	Historical control	1385	1466	1090	1244	1.08	[1.05; 1.10]	6.2%
O'Leary et al. 2019	USA	Patient	RCT	57	80	21	31	1.05	[0.80; 1.39]	4.4%
O'Leary et al. 2019	USA	Patient	RCT	43	62	21	31	1.02	[0.76; 1.37]	4.2%
O'Leary et al. 2019	USA	Patient	RCT	57	80	43	62	1.03	[0.83; 1.27]	5.0%
Omer et al. 2022	USA	Systems, Provider, & Patient	RCT	424	523	425	525	1.00	[0.94; 1.06]	6.1%
Omer et al. 2022	USA	Patient	RCT	414	520	425	525	0.98	[0.93; 1.04]	6.1%
Payakachat et al. 2016	USA	Patient	Historical control	66	135	65	144	1.08	[0.84; 1.39]	4.6%
**Random effects model**								**1.22**	**[0.93; 1.62]**	**41.1%**
Heterogeneity: $I^2 = 79.1\%$, $\tau^2 = 0.1655$, $p < 0.001$										
**Random effects model**								**1.20**	**[1.08; 1.33]**	**100.0%**

Heterogeneity: $I^2 = 67.1\%$, $\tau^2 = 0.0471$, $p < 0.001$
Test for subgroup differences: $\chi^2_1 = 0.01$, df = 1 ($p = 0.9219$)

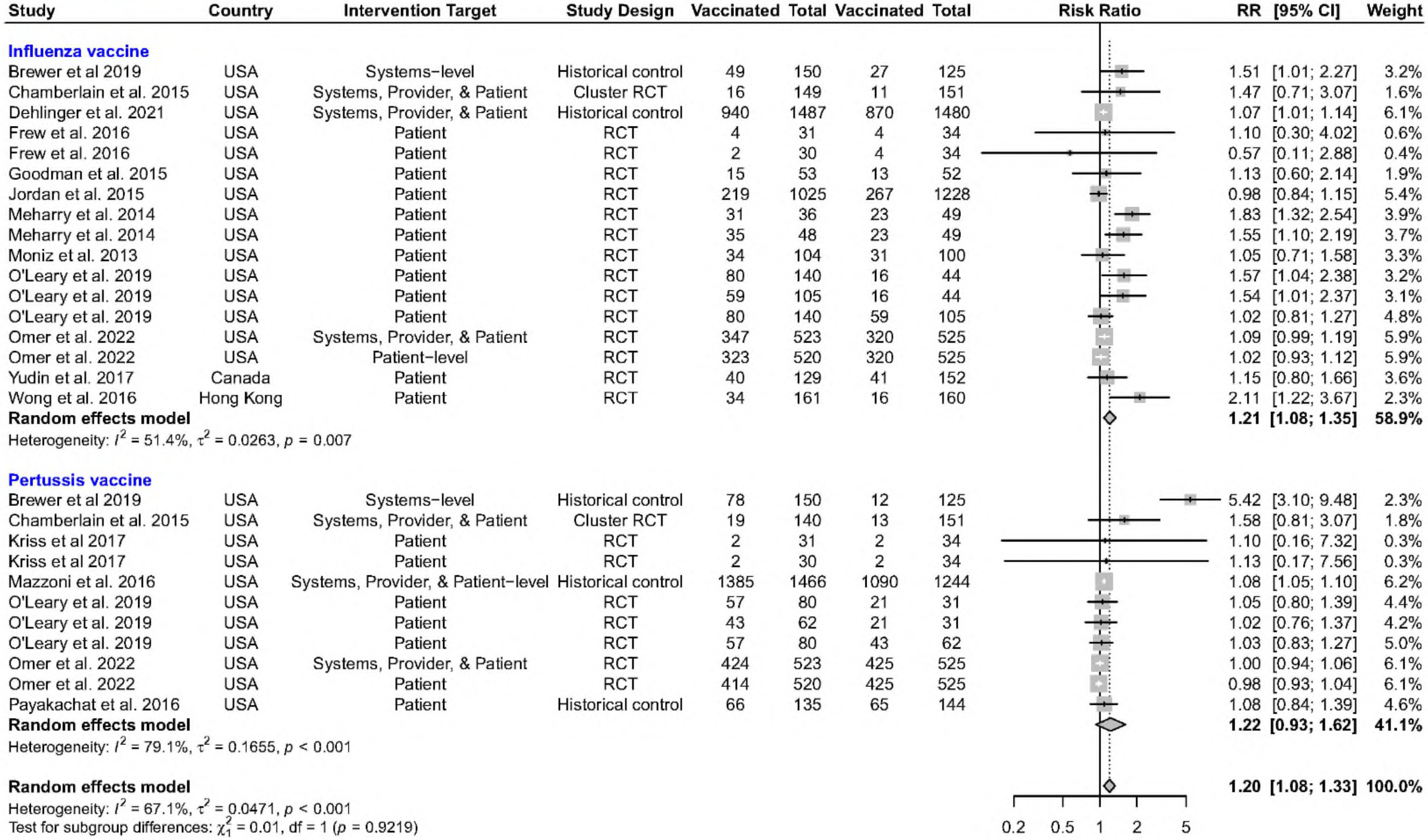

Figure S3. Funnel plot of patient-level interventions to increase uptake of recommended vaccines during pregnancy.

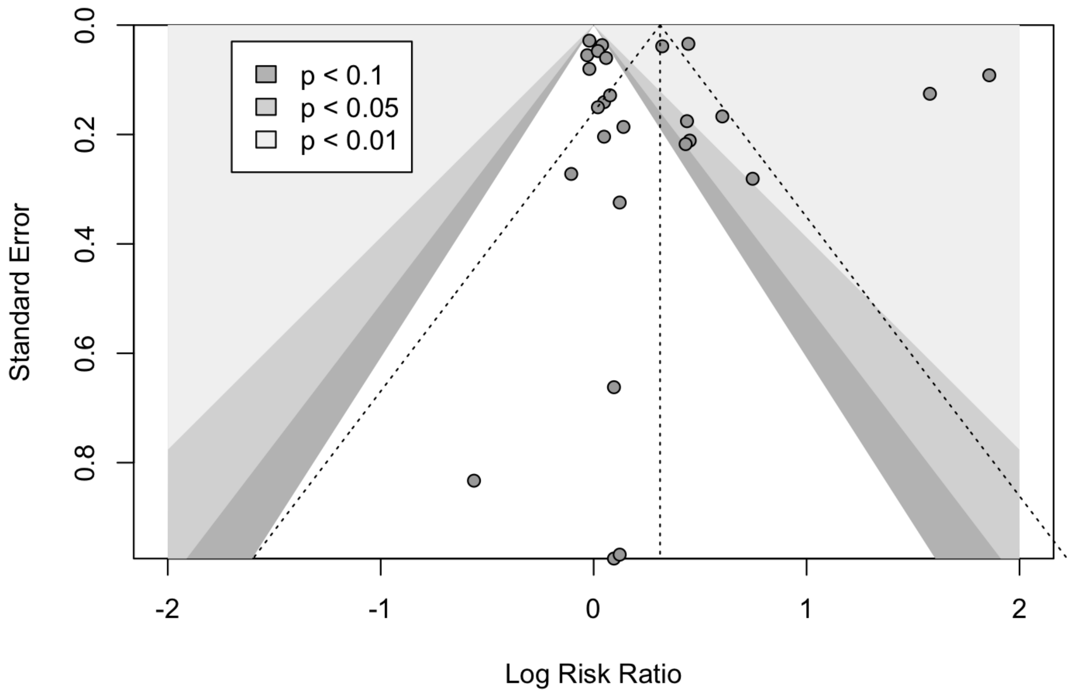

Figure S4. Funnel plot of patient education intervention to increase uptake of recommended vaccines during pregnancy.

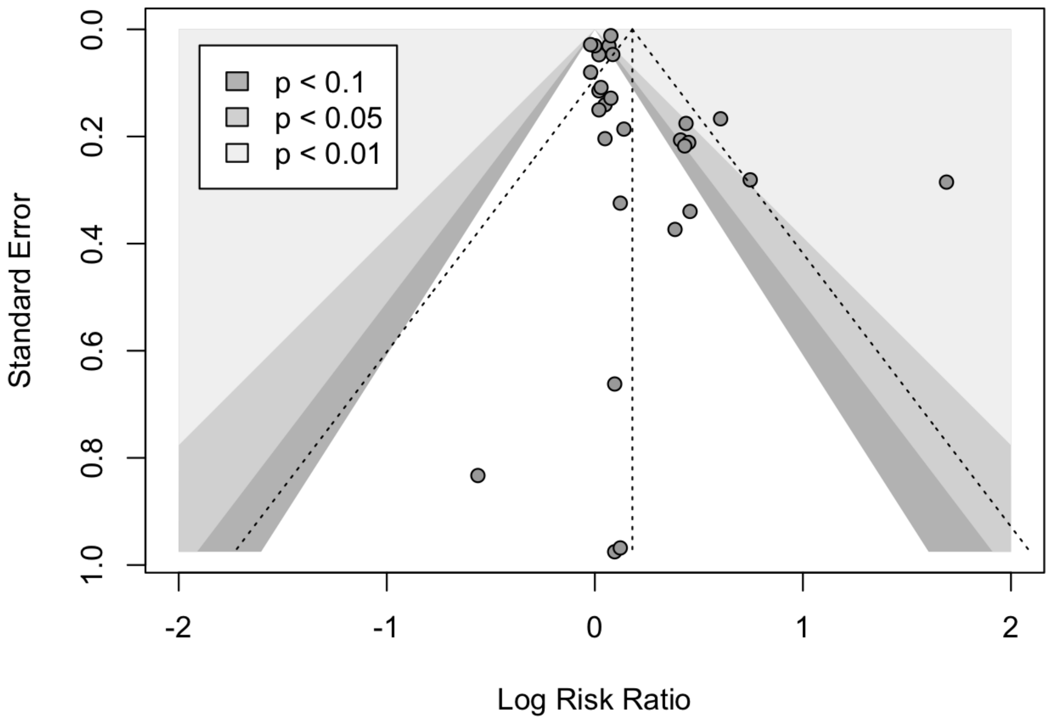

Figure S5. Effect of provider education interventions to increase the uptake of recommended vaccines during pregnancy, by recommended vaccine.

Vaccine Target / Study	Country	Intervention Target	Study Design	Intervention Group Vaccinated	Intervention Group Total	Control Group Vaccinated	Control Group Total	RR [95% CI]	Weight
**Influenza vaccine**									
Brewer et al. 2019	USA	Systems	Historical control	49	150	27	125	1.51 [1.01; 2.27]	5.6%
Chamberlain et al. 2015	USA	Systems, Provider, & Patient	Cluster RCT	16	149	11	151	1.47 [0.71; 3.07]	3.1%
Dehlinger et al. 2021	USA	Systems, Provider, & Patient	Historical control	940	1487	870	1480	1.07 [1.01; 1.14]	8.4%
Mouzoon et al. 2010	USA	Systems & Practice	Historical control	633	1945	427	2023	1.54 [1.39; 1.71]	8.2%
Omer et al. 2022	USA	Systems, Provider, & Patient	RCT	347	523	320	525	1.09 [0.99; 1.19]	8.3%
Omer et al. 2022	USA	Systems & Provider	RCT	327	524	320	525	1.02 [0.93; 1.13]	8.3%
Spina et al. 2020	USA	Systems & Provider-level	Historical control	288	443	250	446	1.16 [1.04; 1.29]	8.2%
**Random effects model**								**1.19 [1.04; 1.36]**	**50.1%**
Heterogeneity: $I^2 = 87\%$, $\tau^2 = 0.0233$, $p < 0.001$									
**Pertussis vaccine**									
Brewer et al. 2019	USA	Systems	Historical control	78	150	12	125	5.42 [3.10; 9.48]	4.2%
Chamberlain et al. 2015	USA	Systems, Provider, & Patient	Cluster RCT	19	140	13	151	1.58 [0.81; 3.07]	3.5%
Mazzoni et al. 2016	USA	Systems, Provider, & Patient-level	Historical control	1385	1466	1090	1244	1.08 [1.05; 1.10]	8.5%
Omer et al. 2022	USA	Systems, Provider, & Patient	RCT	424	523	425	525	1.00 [0.94; 1.06]	8.4%
Omer et al. 2022	USA	Systems & Provider	RCT	399	524	425	525	0.94 [0.88; 1.00]	8.4%
Spina et al. 2020	USA	Systems & Provider-level	Historical control	372	443	343	446	1.09 [1.02; 1.17]	8.4%
**Random effects model**								**1.39 [0.85; 2.27]**	**41.4%**
Heterogeneity: $I^2 = 91\%$, $\tau^2 = 0.3483$, $p < 0.001$									
**Tetanus toxoid vaccine**									
Woolley et al. 2018	Phillipines	Provider	Geographic control	306	333	469	494	0.97 [0.93; 1.00]	8.5%
**Random effects model**								**1.21 [1.03; 1.42]**	**100.0%**
Heterogeneity: $I^2 = 90\%$, $\tau^2 = 0.0805$, $p < 0.001$									
Test for subgroup differences: $\chi^2_2 = 10.31$, df = 2 (p<0.001)									

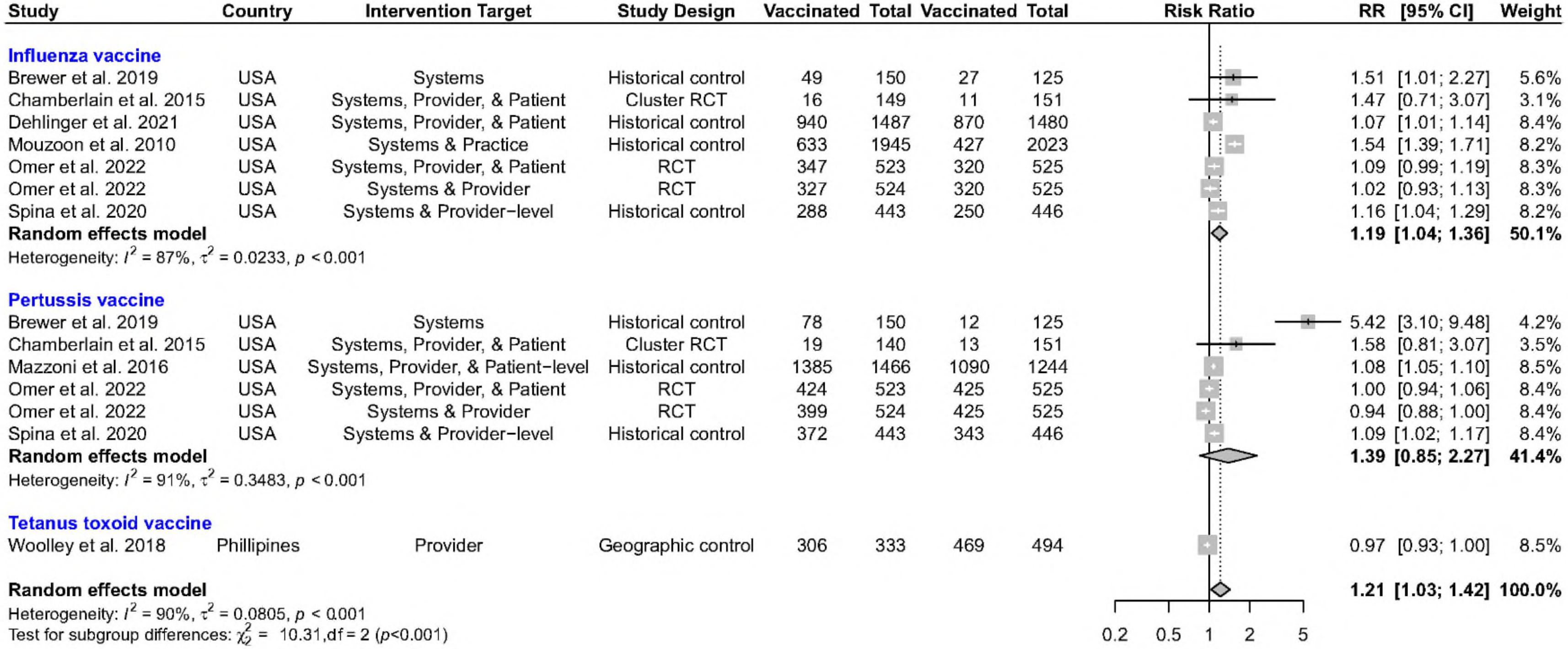

Figure S6. Effect of provider reminder interventions to increase the uptake of recommended vaccines during pregnancy, by recommended vaccine.

Vaccine Target				Intervention Group		Control Group				
**Study**	**Country**	**Intervention Target**	**Study Design**	**Vaccinated**	**Total**	**Vaccinated**	**Total**	**RR**	**[95%CI]**	**Weight**
**Tetanus toxoid vaccine**										
Balakrishnan et al. 2016	India	Systems	Historical control	15785	19880	14731	19880	1.07	[1.06; 1.08]	34.8%
**Influenza vaccine**										
Dehlinger et al. 2021	USA	Systems, Provider, & Patient	Historical control	940	1487	870	1480	1.07	[1.01; 1.14]	33.7%
Klatt & Hopp 2012	USA	Provider	Historical control	393	645	267	639	1.46	[1.31; 1.63]	31.5%
**Random effects model**								**1.25**	**[0.92; 1.69]**	**65.2%**
Heterogeneity: $I^2 = 96\%$, $\tau^2 = 0.0463$, $p < 0.001$										
**Random effects model**								**1.18**	**[0.97; 1.44]**	**100.0%**
Heterogeneity: $I^2 = 94\%$, $\tau^2 = 0.0290$, $p < 0.001$										
Test for subgroup differences: $\chi^2_1 = 0.96$, df = 1 ($p = 0.33$)										

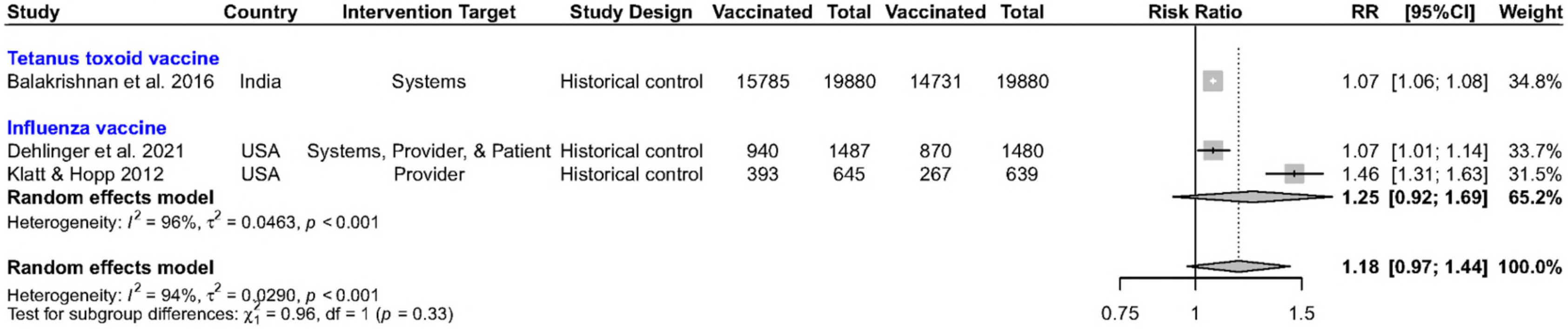

Figure S7. Effect of interventions with immunization champion and enhanced vaccine documentation to increase the uptake of recommended vaccines during pregnancy, by recommended vaccine.

Vaccine Target				Intervention Group		Control Group			
**Study**	**Country**	**Intervention Target**	**Study Design**	**Vaccinated**	**Total**	**Vaccinated**	**Total**	**RR [95% CI]**	**Weight**
**Influenza vaccine**									
Brewer et al. 2019	USA	Systems	Historical control	49	150	27	125	1.51 [1.01; 2.27]	7.2%
Chamberlain et al. 2015	USA	Systems, Provider, & Patient	Cluster RCT	16	149	11	151	1.47 [0.71; 3.07]	4.5%
Mouzoon et al. 2010	USA	Systems & Practice	Historical control	633	1945	427	2023	1.54 [1.39; 1.71]	9.6%
Omer et al. 2022	USA	Systems, Provider, & Patient	RCT	347	523	320	525	1.09 [0.99; 1.19]	9.7%
Omer et al. 2022	USA	Systems & Provider	RCT	327	524	320	525	1.02 [0.93; 1.13]	9.6%
Spina et al. 2020	USA	Systems & Provider	Historical control	288	443	250	446	1.16 [1.04; 1.29]	9.6%
**Random effects model**								**1.22 [1.04; 1.43]**	**50.2%**
Heterogeneity: $I^2 = 87\%$, $\tau^2 = 0.0276$, $p < 0.001$									
**Pertussis vaccine**									
Brewer et al. 2019	USA	Systems	Historical control	78	150	12	125	5.42 [3.10; 9.48]	5.8%
Chamberlain et al. 2015	USA	Systems, Provider, & Patient	Cluster RCT	19	140	13	151	1.58 [0.81; 3.07]	4.9%
Mazzoni et al. 2016	USA	Systems, Provider, & Patient−level	Historical control	1385	1466	1090	1244	1.08 [1.05; 1.10]	9.8%
Omer et al. 2022	USA	Systems, Provider, & Patient	RCT	424	523	425	525	1.00 [0.94; 1.06]	9.8%
Omer et al. 2022	USA	Systems & Provider	RCT	399	524	425	525	0.94 [0.88; 1.00]	9.8%
Spina et al. 2020	USA	Systems & Provider	Historical control	372	443	343	446	1.09 [1.02; 1.17]	9.8%
**Random effects model**								**1.39 [0.85; 2.27]**	**49.8%**
Heterogeneity: $I^2 = 91\%$, $\tau^2 = 0.3483$, $p < 0.001$									
**Random effects model**								**1.27 [1.03; 1.57]**	**100.0%**

Heterogeneity: $I^2 = 90\%$, $\tau^2 = 0.1150$, $p < 0.001$
Test for subgroup differences: $\chi^2_1 = 0.23$, df = 1 ($p = 0.63$)

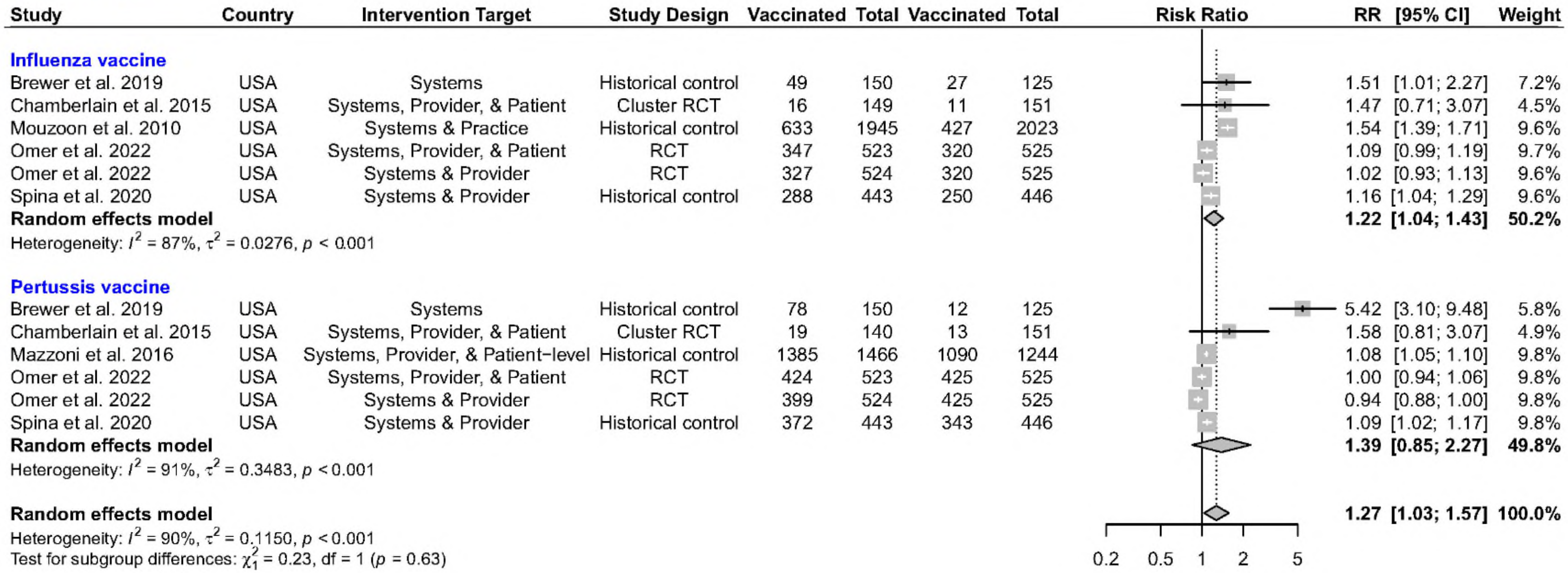

Figure S8. Effect of standing orders and Assessment, Feedback, Incentives, and eXchange (AFIX) programs to increase the uptake of recommended vaccines during pregnancy, by recommended vaccine.

Study	Country	Intervention Target	Study Design	Intervention Group Vaccinated	Intervention Group Total	Control Group Vaccinated	Control Group Total	RR [95% CI]	Weight
**Influenza vaccine**									
Brewer et al. 2019	USA	Systems	Historical control	49	150	27	125	1.51 [1.01; 2.27]	8.2%
Mouzoon et al. 2010	USA	Systems & Practice	Historical control	633	1945	427	2023	1.54 [1.39; 1.71]	10.5%
Omer et al. 2022	USA	Systems, Provider, & Patient	RCT	347	523	320	525	1.09 [0.99; 1.19]	10.6%
Omer et al. 2022	USA	Systems & Provider	RCT	327	524	320	525	1.02 [0.93; 1.13]	10.6%
Spina et al. 2020	USA	Systems & Provider	Historical control	288	443	250	446	1.16 [1.04; 1.29]	10.5%
**Random effects model**								**1.21 [1.03; 1.43]**	**50.5%**
Heterogeneity: $I^2 = 90\%$, $\tau^2 = 0.0288$, $p < 0.001$									
**Pertussis vaccine**									
Brewer et al. 2019	USA	Systems	Historical control	78	150	12	125	5.42 [3.10; 9.48]	6.8%
Mazzoni et al. 2016	USA	Systems, Provider, & Patient-level	Historical control	1385	1466	1090	1244	1.08 [1.05; 1.10]	10.7%
Omer et al. 2022	USA	Systems, Provider, & Patient	RCT	424	523	425	525	1.00 [0.94; 1.06]	10.7%
Omer et al. 2022	USA	Systems & Provider	RCT	399	524	425	525	0.94 [0.88; 1.00]	10.7%
Spina et al. 2020	USA	Systems & Provider	Historical control	372	443	343	446	1.09 [1.02; 1.17]	10.7%
**Random effects model**								**1.37 [0.76; 2.47]**	**49.5%**
Heterogeneity: $I^2 = 92\%$, $\tau^2 = 0.4385$, $p < 0.001$									
**Random effects model**								**1.26 [0.99; 1.60]**	**100.0%**
Heterogeneity: $I^2 = 92\%$, $\tau^2 = 0.1381$, $p < 0.001$									
Test for subgroup differences: $\chi^2_1 = 0.15$, df = 1 ($p = 0.70$)									

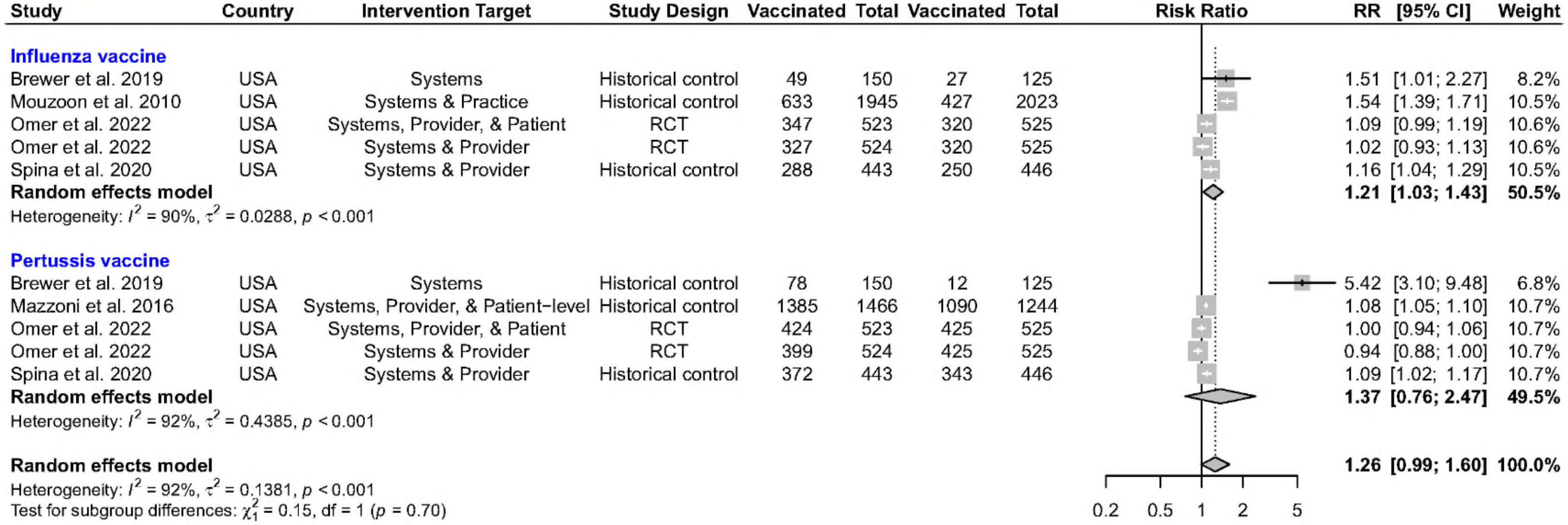

Figure S9. Funnel plot of provider or systems-level interventions to increase uptake of recommended vaccines during pregnancy.

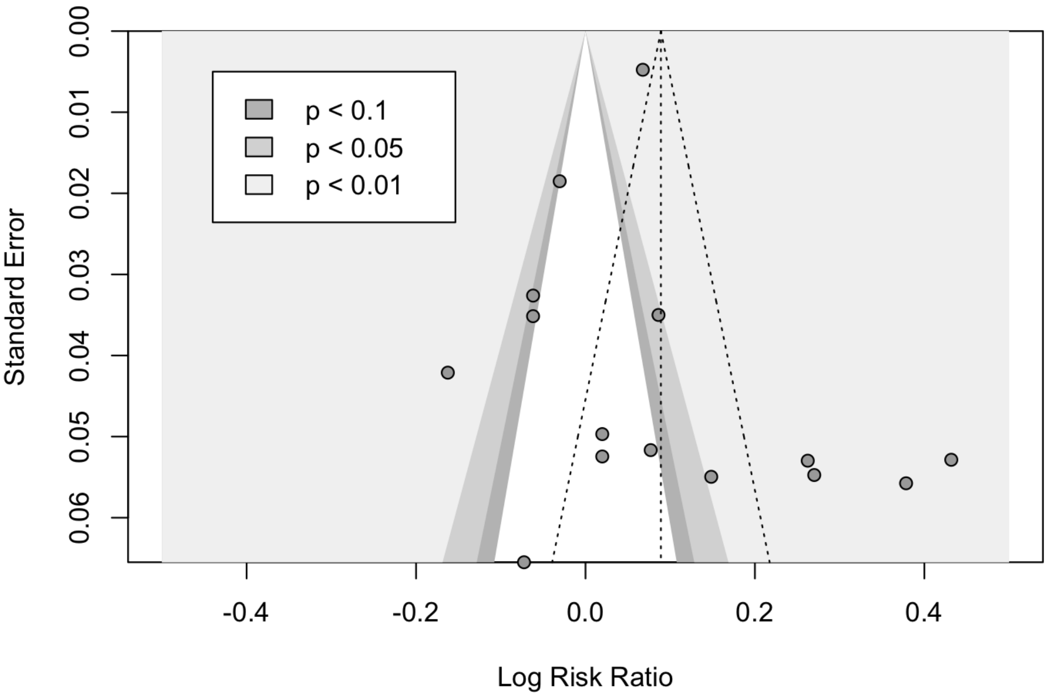

Figure S10. Funnel plot of provider education intervention to increase uptake of recommended vaccines during pregnancy.

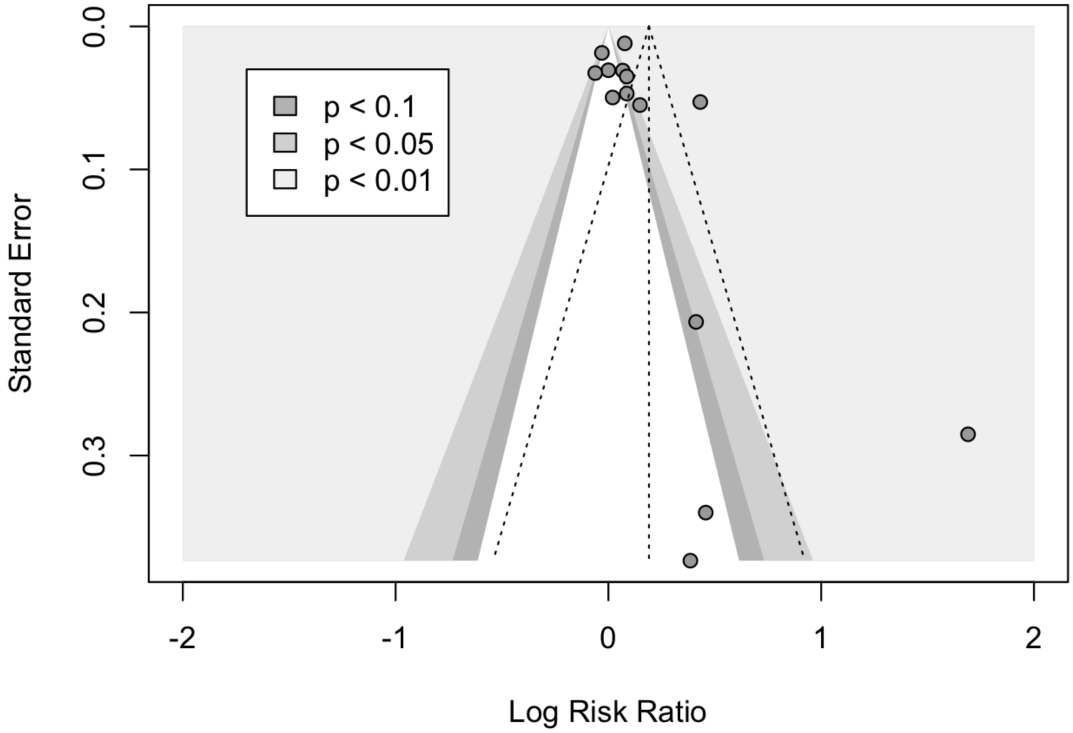

Figure S11. Funnel plot of provider reminder intervention to increase uptake of recommended vaccines during pregnancy.

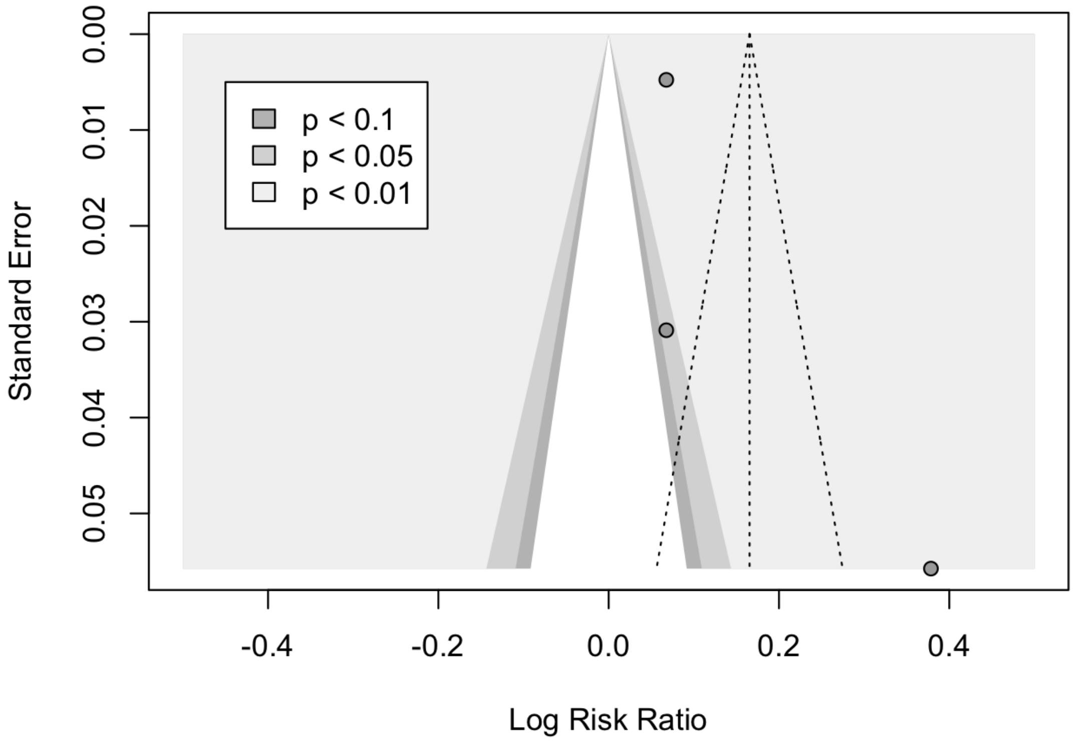

Figure S12. Funnel plot of interventions with immunization champions and enhanced vaccine documentation to increase the uptake of recommended vaccines during pregnancy.

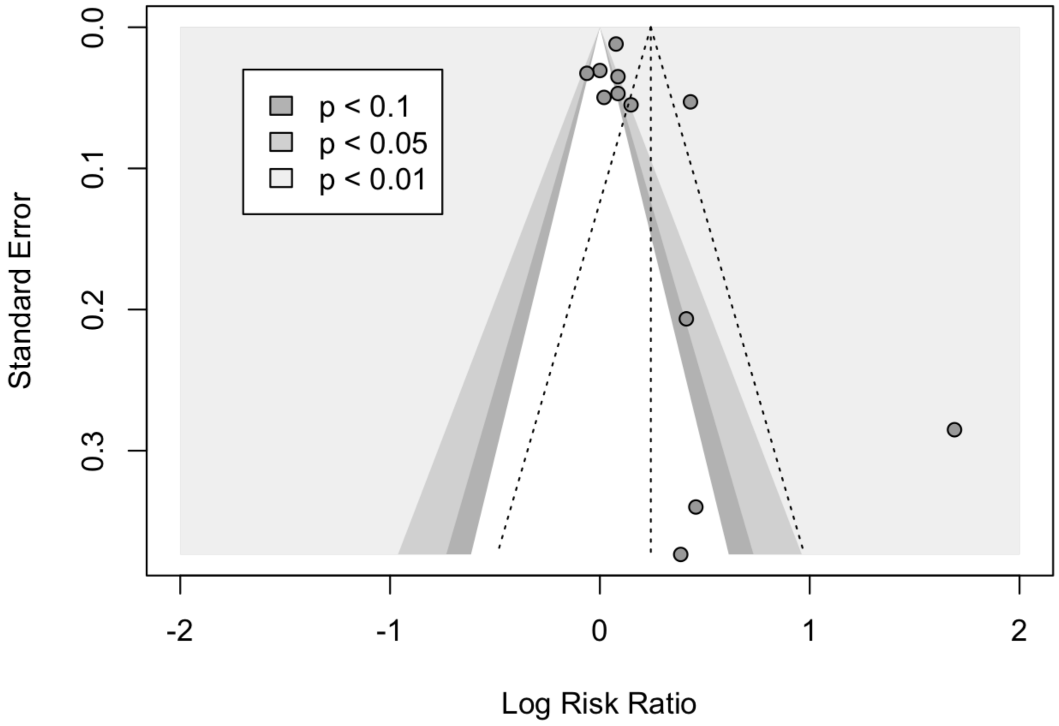

Figure S13. Funnel plot of interventions with standing orders and Assessment, Feedback, Incentives, and eXchange (AFIX) programs to increase the uptake of recommended vaccines during pregnancy.

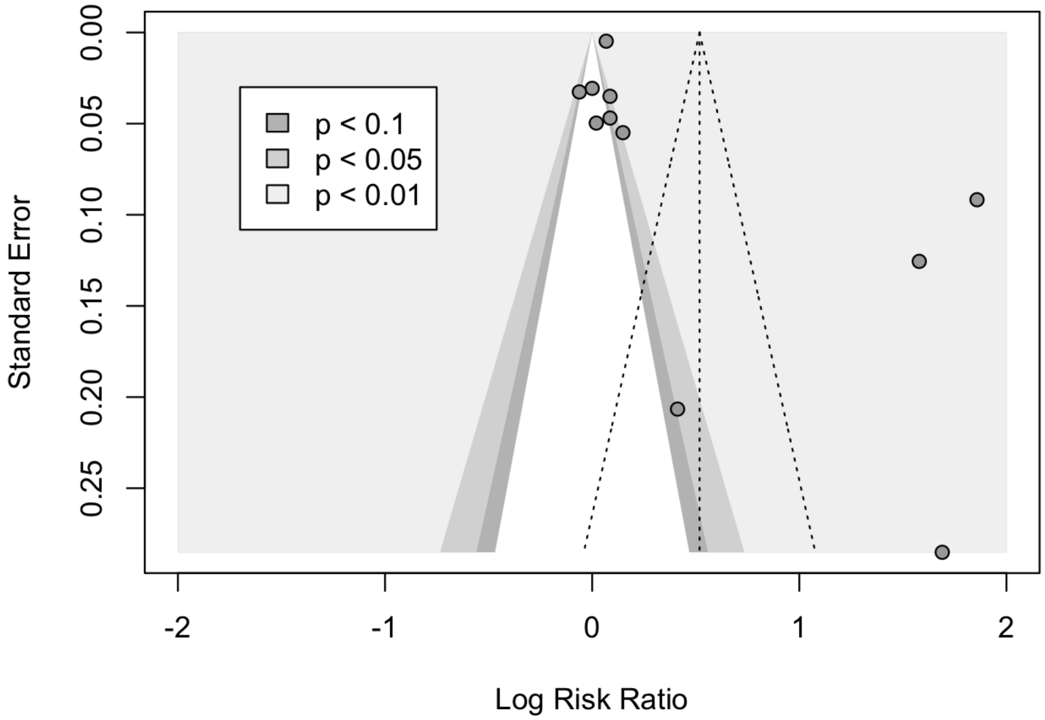

Figure S14. Funnel plot of patient and provider or systems-level interventions to increase uptake of recommended vaccines during pregnancy.

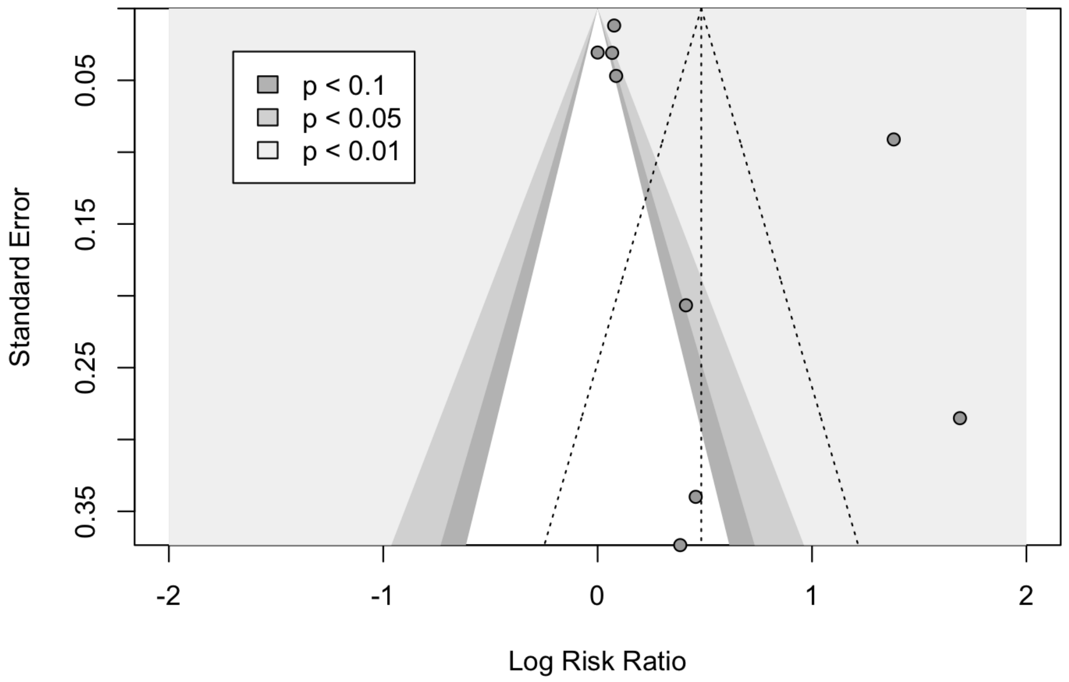

Supplementary Note A. Central Search Strategy: Interventions to increase vaccine uptake during pregnancy

#1 MeSH descriptor: [Pregnancy Trimesters] explode all trees
#2 MeSH descriptor: [Pregnant Women] this term only
#3 MeSH descriptor: [Pregnancy] explode all trees
#4 pregnan* or matern*
#5 #1 or #2 or #3 or #4
#6 MeSH descriptor: [Vaccines] this term only
#7 MeSH descriptor: [COVID-19 Vaccines] explode all trees
#8 MeSH descriptor: [ChAdOx1 nCoV-19] explode all trees
#9 MeSH descriptor: [Tetanus Toxoid] explode all trees
#10 MeSH descriptor: [2019-nCoV Vaccine mRNA-1273] explode all trees
#11 MeSH descriptor: [BNT162 Vaccine] explode all trees
#12 MeSH descriptor: [Pertussis Vaccine] explode all trees
#13 MeSH descriptor: [Influenza Vaccines] explode all trees
#14 MeSH descriptor: [mRNA Vaccines] explode all trees
#15 ((pertussis or influenza or flu or tetanus or diptheria or polio or DPT or DTP or
"DTaP/IPV" or "dTaP/IPV" or
whooping cough or COVID-19* or SARS-CoV-2) near/3 (vaccin* or immunis* or immuniz* or inoculat* or innoculat*
or prevent*))
#16 MeSH descriptor: [Vaccines] explode all trees
#17 MeSH descriptor: [Vaccination Refusal] explode all trees
#18 MeSH descriptor: [Immunity] in all MeSH products
#19 MeSH descriptor: [Whooping Cough] this term only and with qualifier(s): [prevention & control - PC]
#20 MeSH descriptor: [Influenza, Human] this term only and with qualifier(s): [prevention & control - PC]
#21 MeSH descriptor: [Tetanus] this term only and with qualifier(s): [prevention & control - PC]
#22 MeSH descriptor: [COVID-19] this term only and with qualifier(s): [prevention & control - PC]
#23 MeSH descriptor: [Diphtheria] this term only and with qualifier(s): [prevention & control - PC]
#24 MeSH descriptor: [Poliomyelitis] this term only and with qualifier(s): [prevention & control - PC]
#25 ((maternal or pregnan*) near/3 (vaccin* or immunis* or immuniz* or inoculat* or innoculat*))
#26 ((vaccin* or immunis* or immuniz* or inoculat* or innoculat*) near/3 (refus* or hesitan* or uptake* or
accept*))
#27 #6 or #7 or #8 or #9 or #10 or #11 or #12 or #13 or #14 or #15 or #16 or #17 or #18 or #19 or #20 or #21 or
#22 or #23 or #24 or #25 or #26
28 #5 and #27
Search run on 4 July 2023 = 2,109

Supplementary Note B. CINAHL Search Strategy: Interventions to increase vaccine uptake during pregnancy

1 (MH "Pregnancy Trimesters+")
2 (MH Pregnancy+) OR (MH "Pregnant Women")
3 ((TI pregnan* OR AB pregnan*) OR (TI matern* OR AB matern*)) ,kw,kf.
4 S1 OR S2 OR S3
5 (MH Vaccines)
6 (MH "COVID-19 Vaccines+")
7 (MH "Tetanus Toxoid+")
8 (MH "Pertussis Vaccine+")
9 (MH "Influenza Vaccines")
10 (MH "mRNA Vaccines+")
11 (((TI pertussis OR AB pertussis) OR (TI influenza OR AB influenza) OR (TI flu OR AB flu) OR (TI tetanus
OR AB
tetanus) OR (TI diptheria OR AB diptheria) OR (TI polio OR AB polio) OR (TI DPT OR AB DPT) OR (TI DTP
OR AB DTP)
OR (TI DTaP/IPV OR AB DTaP/IPV) OR (TI dTaP/IPV OR AB dTaP/IPV) OR (TI "whooping
cough" OR AB "whooping
cough") OR (TI COVID-19* OR AB COVID-19*) OR (TI SARS-CoV-2 OR AB SARS-CoV-2)) N3 ((TI
vaccin* OR AB vaccin*)
OR (TI immunis* OR AB immunis*) OR (TI immuniz* OR AB immuniz*) OR (TI inoculat* OR AB inoculat*)
OR (TI
innoculat* OR AB innoculat*) OR (TI prevent* OR AB prevent*))) ,kw,kf.
12 (MH Vaccination+)
13 (MH "Vaccination Refusal+")
14 (MH Immunization+)
15 (MH "Whooping Cough")
16 (MH "Influenza, Human")
17 (MH Tetanus)
18 (MH COVID-19)
19 (MH Diphtheria)
20 (MH Poliomyelitis)
21 (((TI maternal OR AB maternal) OR (TI pregnan* OR AB pregnan*)) N3 ((TI vaccin* OR AB vaccin*) OR
(TI immunis*
OR AB immunis*) OR (TI immuniz* OR AB immuniz*) OR (TI inoculat* OR AB inoculat*) OR (TI innoculat*
OR AB
innoculat*))) ,kw,kf.
22 (((TI vaccin* OR AB vaccin*) OR (TI immunis* OR AB immunis*) OR (TI immuniz* OR AB immuniz*) OR
(TI
inoculat* OR AB inoculat*) OR (TI innoculat* OR AB innoculat*)) N3 ((TI refus* OR AB refus*) OR (TI
hesitan* OR AB

hesitan*) OR (TI uptake* OR AB uptake*) OR (TI accept* OR AB accept*))) ,kw,kf.
23 S5 OR S6 OR S7 OR S8 OR S9 OR S10 OR S11 OR S12 OR S13 OR S14 OR S15 OR S16 OR S17 OR S18 OR S19 OR S20
OR S21 OR S22
24 (PT "randomized controlled trial")
25 (PT "controlled clinical trial")
26 (AB placebo)
27 "Drug Therapy"
28 (AB randomly)
29 (TI trial)
30 (AB groups)
31 (((AB singl*) OR (AB doubl*) OR (AB trebl*) OR (AB tripl*)) W1 ((AB mask*) OR (AB blind*) OR (AB dumm*)))
32 ((AB randomi?ed) OR (AB randomi?ation))
33 ((AB quasi-random*) OR (AB quasi-experiment*) OR (AB "stepped wedge"))
34 S24 OR S25 OR S26 OR S27 OR S28 OR S29 OR S30 OR S31 OR S32 OR S33
35 ((MH animals) NOT ((MH humans) AND (MH animals)))
36 S34 NOT S35
37 S4 AND S23 AND S36

Search run on 5 July 2023 = 774

Supplementary Note C. Embase Search Strategy: Interventions to increase vaccine uptake during pregnancy

1. exp pregnancy/
2. pregnant woman/
3. (pregnan* or matern*).ti,ab,kw,kf.
4. 1 or 2 or 3
5. vaccine/
6. exp SARS-CoV-2 vaccine/
7. exp tetanus toxoid/
8. exp pertussis vaccine/
9. influenza vaccine/
10. exp RNA vaccine/
11. ((pertussis or influenza or flu or tetanus or diptheria or polio or DPT or DTP or
"DTaP/IPV" or "dTaP/IPV" or
whooping cough or COVID-19* or SARS-CoV-2) adj3 (vaccin* or immunis* or immuniz* or inoculat* or innoculat* or
prevent*)).ti,ab,kw,kf.
12. exp vaccination/
13. exp vaccination refusal/
14. exp immunization/
15. pertussis/pc [Prevention]
16. influenza/pc [Prevention]
17. tetanus/pc [Prevention]
18. coronavirus disease 2019/pc [Prevention]
19. diphtheria/pc [Prevention]
20. poliomyelitis/pc [Prevention]
21. ((maternal or pregnan*) adj3 (vaccin* or immunis* or immuniz* or inoculat* or innoculat*)).ti,ab,kw,kf.
22. ((vaccin* or immunis* or immuniz* or inoculat* or innoculat*) adj3 (refus* or hesitan* or uptake* or accept*)).ti,ab,kw,kf.
23. 5 or 6 or 7 or 8 or 9 or 10 or 11 or 12 or 13 or 14 or 15 or 16 or 17 or 18 or 19 or 20 or 21 or 22
24. crossover procedure/
25. double-blind procedure/
26. randomized controlled trial/
27. single-blind procedure/
28. random*.mp.
29. factorial*.mp.
30. (crossover* or cross over* or cross-over*).mp.
31. placebo*.mp.
32. assign*.mp.
33. allocat*.mp.

34. volunteer*.mp.
35. ((singl* or doubl* or trebl* or tripl*) adj (mask* or blind* or dumm*)).ab.
36. (quasi-random* or quasi-experiment* or stepped wedge).ab.
37. 24 or 25 or 26 or 27 or 28 or 29 or 30 or 31 or 32 or 33 or 34 or 35 or 36
38. 4 and 23 and 37

Search run on 30 June 2023 = 3,242

Supplementary Note D. Medline Search Strategy: Interventions to increase vaccine uptake during pregnancy

1. exp Pregnancy Trimesters/
2. exp Pregnancy/ or Pregnant Women/
3. (pregnan* or matern*).ti,ab,kw,kf.
4. 1 or 2 or 3
5. Vaccines/
6. exp COVID-19 Vaccines/
7. exp Tetanus Toxoid/
8. exp Pertussis Vaccine/
9. Influenza Vaccines/
10. exp mRNA Vaccines/
11. ((pertussis or influenza or flu or tetanus or diptheria or polio or DPT or DTP or "DTaP/IPV" or "dTaP/IPV" or whooping cough or COVID-19* or SARS-CoV-2) adj3 (vaccin* or immunis* or immuniz* or inoculat* or innoculat* or prevent*)).ti,ab,kw,kf.
12. exp Vaccination/
13. exp Vaccination Refusal/
14. exp Immunization/
15. Whooping Cough/pc [Prevention & Control]
16. Influenza, Human/pc [Prevention & Control]
17. Tetanus/pc [Prevention & Control]
18. COVID-19/pc [Prevention & Control]
19. Diphtheria/pc [Prevention & Control]
20. Poliomyelitis/pc [Prevention & Control]
21. ((maternal or pregnan*) adj3 (vaccin* or immunis* or immuniz* or inoculat* or innoculat*)).ti,ab,kw,kf.
22. ((vaccin* or immunis* or immuniz* or inoculat* or innoculat*) adj3 (refus* or hesitan* or uptake* or accept*)).ti,ab,kw,kf.
23. 5 or 6 or 7 or 8 or 9 or 10 or 11 or 12 or 13 or 14 or 15 or 16 or 17 or 18 or 19 or 20 or 21 or 22
24. randomized controlled trial.pt.
25. controlled clinical trial.pt.
26. placebo.ab.
27. drug therapy.fs.
28. randomly.ab.
29. trial.ti.
30. groups.ab.
31. ((singl* or doubl* or trebl* or tripl*) adj (mask* or blind* or dumm*)).ab.
32. (randomi#ed or randomi#ation).ab.
33. (quasi-random* or quasi-experiment* or stepped wedge).ab.
34. 24 or 25 or 26 or 27 or 28 or 29 or 30 or 31 or 32 or 33
35. (animals not (humans and animals)).sh.

36. 34 not 35
37. 4 and 23 and 36

Search run through 3 July 2023 = 1759

Supplementary Note E. SCOPUS Search Strategy: Interventions to increase vaccine uptake during pregnancy

1 INDEXTERMS("Pregnancy Trimesters")
2 INDEXTERMS(Pregnancy) OR INDEXTERMS("Pregnant Women")
3 TITLE-ABS(pregnan* OR matern*) ,kw,kf.
4 #1 OR #2 OR #3
5 INDEXTERMS(Vaccines)
6 INDEXTERMS("COVID-19 Vaccines")
7 INDEXTERMS("Tetanus Toxoid")
8 INDEXTERMS("Pertussis Vaccine")
9 INDEXTERMS("Influenza Vaccines")
10 INDEXTERMS("mRNA Vaccines")
11 TITLE-ABS((pertussis OR influenza OR flu OR tetanus OR diptheria OR polio OR DPT OR DTP OR DTaP/IPV OR
dTaP/IPV OR "whooping cough" OR COVID-19* OR SARS-CoV-2) W/3 (vaccin* OR immunis* OR immuniz* OR
inoculat* OR innoculat* OR prevent*)) ,kw,kf.
12 INDEXTERMS(Vaccination)
13 INDEXTERMS("Vaccination Refusal")
14 INDEXTERMS(Immunization)
15 INDEXTERMS("Whooping Cough")
16 INDEXTERMS("Influenza, Human")
17 INDEXTERMS(Tetanus)
18 INDEXTERMS(COVID-19)
19 INDEXTERMS(Diphtheria)
20 INDEXTERMS(Poliomyelitis)
21 TITLE-ABS((maternal OR pregnan*) W/3 (vaccin* OR immunis* OR immuniz* OR inoculat* OR innoculat*))
,kw,kf.
22 TITLE-ABS((vaccin* OR immunis* OR immuniz* OR inoculat* OR innoculat*) W/3 (refus* OR hesitan* OR uptake*
OR accept*)) ,kw,kf.
23 #5 OR #6 OR #7 OR #8 OR #9 OR #10 OR #11 OR #12 OR #13 OR #14 OR #15 OR #16 OR #17 OR #18 OR #19 OR
#20 OR #21 OR #22
24 DOCTYPE("randomized controlled trial")
25 DOCTYPE("controlled clinical trial")
26 ABS(placebo)
27 "Drug Therapy"
28 ABS(randomly)
29 TITLE(trial)

30 ABS(groups)
31 ABS((singl* OR doubl* OR trebl* OR tripl*) W/1 (mask* OR blind* OR dumm*))
32 ABS(randomi?ed OR randomi?ation)
33 ABS(quasi-random* OR quasi-experiment* OR "stepped wedge")
34 #24 OR #25 OR #26 OR #27 OR #28 OR #29 OR #30 OR #31 OR #32 OR #33
35 #4 AND #23 AND #34

Search run on 4 July 2023 = 6,488

Supplementary Note F. PRISMA checklist for systematic reviews and meta-analyses.

Section and Topic	Item #	Checklist item	Location where item is reported
**TITLE**			
Title	1	Identify the report as a systematic review.	Title
**ABSTRACT**			
Abstract	2	See the PRISMA 2020 for Abstracts checklist.	Abstract
**INTRODUCTION**			
Rationale	3	Describe the rationale for the review in the context of existing knowledge.	Page 4
Objectives	4	Provide an explicit statement of the objective(s) or question(s) the review addresses.	Page 4
**METHODS**			
Eligibility criteria	5	Specify the inclusion and exclusion criteria for the review and how studies were grouped for the syntheses.	Page 5
Information sources	6	Specify all databases, registers, websites, organisations, reference lists and other sources searched or consulted to identify studies. Specify the date when each source was last searched or consulted.	Page 5
Search strategy	7	Present the full search strategies for all databases, registers and websites, including any filters and limits used.	Web Appendix A-E
Selection process	8	Specify the methods used to decide whether a study met the inclusion criteria of the review, including how many reviewers screened each record and each report retrieved, whether they worked independently, and if applicable, details of automation tools used in the process.	Page 6
Data collection process	9	Specify the methods used to collect data from reports, including how many reviewers collected data from each report, whether they worked independently, any processes for obtaining or confirming data from study investigators, and if applicable, details of automation tools used in the process.	Page 6
Data items	10a	List and define all outcomes for which data were sought. Specify whether all results that were compatible with each outcome domain in each study were sought (e.g. for all measures, time points, analyses), and if not, the methods used to decide which results to collect.	Page 5
	10b	List and define all other variables for which data were sought (e.g. participant and intervention characteristics, funding sources). Describe any assumptions made about any missing or unclear information.	Page 6
Study risk of bias assessment	11	Specify the methods used to assess risk of bias in the included studies, including details of the tool(s) used, how many reviewers assessed each study and whether they worked independently, and if applicable, details of automation tools used in the process.	Page 7
Effect measures	12	Specify for each outcome the effect measure(s) (e.g. risk ratio, mean difference) used in the synthesis or presentation of results.	Page 7
Synthesis methods	13a	Describe the processes used to decide which studies were eligible for each synthesis (e.g. tabulating the study intervention characteristics and comparing against the planned groups for each synthesis (item #5)).	Page 7
	13b	Describe any methods required to prepare the data for presentation or synthesis, such as handling of missing summary statistics, or data conversions.	Page 7

Section and Topic	Item #	Checklist item	Location where item is reported
	13c	Describe any methods used to tabulate or visually display results of individual studies and syntheses.	Page 7
	13d	Describe any methods used to synthesize results and provide a rationale for the choice(s). If meta-analysis was performed, describe the model(s), method(s) to identify the presence and extent of statistical heterogeneity, and software package(s) used.	Page 7
	13e	Describe any methods used to explore possible causes of heterogeneity among study results (e.g. subgroup analysis, meta-regression).	Page 7
	13f	Describe any sensitivity analyses conducted to assess robustness of the synthesized results.	NA
Reporting bias assessment	14	Describe any methods used to assess risk of bias due to missing results in a synthesis (arising from reporting biases).	Page 7
Certainty assessment	15	Describe any methods used to assess certainty (or confidence) in the body of evidence for an outcome.	Page 7
**RESULTS**			
Study selection	16a	Describe the results of the search and selection process, from the number of records identified in the search to the number of studies included in the review, ideally using a flow diagram.	Page 8
	16b	Cite studies that might appear to meet the inclusion criteria, but which were excluded, and explain why they were excluded.	Table S1
Study characteristics	17	Cite each included study and present its characteristics.	Table 1
Risk of bias in studies	18	Present assessments of risk of bias for each included study.	Table 2 and 3
Results of individual studies	19	For all outcomes, present, for each study: (a) summary statistics for each group (where appropriate) and (b) an effect estimate and its precision (e.g. confidence/credible interval), ideally using structured tables or plots.	Figures 3, 4 and 5
Results of syntheses	20a	For each synthesis, briefly summarise the characteristics and risk of bias among contributing studies.	Page 9, Table 4
	20b	Present results of all statistical syntheses conducted. If meta-analysis was done, present for each the summary estimate and its precision (e.g. confidence/credible interval) and measures of statistical heterogeneity. If comparing groups, describe the direction of the effect.	Figures 3, 4 and 5
	20c	Present results of all investigations of possible causes of heterogeneity among study results.	Figures S2, S4, S5-S8
	20d	Present results of all sensitivity analyses conducted to assess the robustness of the synthesized results.	NA
Reporting biases	21	Present assessments of risk of bias due to missing results (arising from reporting biases) for each synthesis assessed.	Figure S3, S4, and S9-14
Certainty of evidence	22	Present assessments of certainty (or confidence) in the body of evidence for each outcome assessed.	Table 4
**DISCUSSION**			

Section and Topic	Item #	Checklist item	Location where item is reported
Discussion	23a	Provide a general interpretation of the results in the context of other evidence.	Page 14-15
	23b	Discuss any limitations of the evidence included in the review.	Page 17-18
	23c	Discuss any limitations of the review processes used.	Page 17-18
	23d	Discuss implications of the results for practice, policy, and future research.	Page 18
**OTHER INFORMATION**			
Registration and protocol	24a	Provide registration information for the review, including register name and registration number, or state that the review was not registered.	Page 4
	24b	Indicate where the review protocol can be accessed, or state that a protocol was not prepared.	Page 4
	24c	Describe and explain any amendments to information provided at registration or in the protocol.	NA
Support	25	Describe sources of financial or non-financial support for the review, and the role of the funders or sponsors in the review.	Funding Statement
Competing interests	26	Declare any competing interests of review authors.	Conflict of interest statement
Availability of data, code and other materials	27	Report which of the following are publicly available and where they can be found: template data collection forms; data extracted from included studies; data used for all analyses; analytic code; any other materials used in the review.	Data availability statement

From: Page MJ, McKenzie JE, Bossuyt PM, Boutron I, Hoffmann TC, Mulrow CD, et al. The PRISMA 2020 statement: an updated guideline for reporting systematic reviews. BMJ 2021;372:n71. doi: 10.1136/bmj.n71
